# Metabolite profiling of peripheral blood plasma in pigs in early postnatal life fed whole bovine, caprine or ovine milk

**DOI:** 10.3389/fnut.2023.1242301

**Published:** 2023-09-26

**Authors:** Ankita Jena, Carlos A. Montoya, Karl Fraser, Caroline Giezenaar, Wayne Young, Jane A. Mullaney, Ryan N. Dilger, Debashree Roy, Warren C. McNabb, Nicole C. Roy

**Affiliations:** ^1^Riddet Institute, Massey University, Palmerston North, New Zealand; ^2^School of Food and Advanced Technology, College of Sciences, Massey University, Palmerston North, New Zealand; ^3^AgResearch, Palmerston North, New Zealand; ^4^High-Value Nutrition National Science Challenge, Auckland, New Zealand; ^5^Food Experience and Sensory Testing (FEAST) Laboratory, School of Food and Advanced Technology, Massey University, Palmerston North, New Zealand; ^6^Department of Animal Sciences, University of Illinois, Urbana, IL, United States; ^7^Department of Human Nutrition, University of Otago, Dunedin, New Zealand

**Keywords:** metabolomics, early life, nutrition, ruminant milk, pigs, plasma, infant, lipids

## Abstract

Ruminants’ milk is commonly used for supplying nutrients to infants when breast milk is unavailable or limited. Previous studies have highlighted the differences between ruminants’ milk composition, digestion, absorption, and fermentation. However, whether consuming different ruminants’ milk impact the appearance of the circulatory blood metabolites in the early postnatal life is not well understood. The analysis conducted here aimed to determine the effect of feeding exclusively whole milk from bovine, caprine or ovine species to pigs, approximately 7 days-old for 15 days, on circulatory blood plasma metabolites. Relative intensities of plasma metabolites were detected using a liquid chromatography-mass spectrometry based metabolomic approach. Seven polar and 83 non-polar (lipids) metabolites in plasma were significantly different (false discovery rate < 0.05) between milk treatments. These included polar metabolites involved in amino acid metabolism and lipids belonging to phosphatidylcholine, lysophosphatidylcholine, sphingomyelin, and triglycerides. Compared to the caprine or bovine milk group, the relative intensities of polar metabolites and unsaturated triglycerides were higher in the peripheral circulation of the ovine milk group. In contrast, relative intensities of saturated triglycerides and phosphatidylcholine were higher in the bovine milk group compared to the ovine or caprine milk group. In addition, correlations were identified between amino acid and lipid intake and their appearance in peripheral blood circulation. The results highlighted that consuming different ruminants’ milk influences the plasma appearance of metabolites, especially lipids, that may contribute to early postnatal life development in pigs.

## 1. Introduction

During the early postnatal years of life, body development occurs rapidly, involving events like the maturation of the tissular structure and function of the gut, the establishment of the gut microbiota, remodelling of the immune system, refinement of brain neural circuits, and establishment of cognitive abilities. Human breast milk or infant formula is one of the factors influencing the developmental events during this period. Human milk is the best nutrient source for infant development. However, formula feeding plays a crucial role in satisfying the nutrient requirements in situations where breastfeeding is either limited or unavailable. Infant formulas are predominantly made with bovine milk. However, non-bovine milk (e.g., caprine and ovine) are also increasingly used due to their hypo-allergenic properties and easier digestion than bovine milk (1–4).

Nutrient composition (5–8) and physiochemical properties (1, 5) between bovine, caprine, and ovine milk differ. Ovine milk has a higher concentration of macronutrients (lipids and proteins) and micronutrients (Ca, P) than bovine or caprine milk (5, 9). Additionally, caprine and ovine casein micelle diameters are greater than bovine milk whereas fat globule diameters are smaller than those in bovine milk (1, 5). Milk composition within the same species also varies considerably due to milking season, breed, type of feed and climate (5, 10).

Milk is largely digested in the stomach and small intestine. The released nutrients are absorbed in the small intestine and metabolised in host tissues. A pig study by Roy et al. (11) showed that the gastric emptying rate of proteins and lipids differed across bovine, caprine and ovine milk, suggesting that this might lead to different absorption in the small intestine and, therefore, the appearance of these nutrients in the peripheral blood circulation across milk treatments.

Milk also contains nutrients like oligosaccharides known to escape digestion in the small intestine and used by the colonic gut microbiota (12–14). Digested ruminant milk that were dialysed to remove digested molecules and then added as a substrate to batch cultures using a faecal inoculum from infants differentially altered the cultured microbial composition depending on the type of milk (15, 16). Fermentation of the remaining undigested material from bovine and ovine milk also resulted in different concentrations of microbial metabolites (e.g., short-chain fatty acids) between milk treatments (15). Thus, it could be expected that host and microbial activities related to the consumption of milk types in early postnatal life might lead to the production of different metabolites involved in signalling, energy conversion, neurotransmission, and cofactor activity, which could influence the development in the first years of postnatal life. For instance, a longitudinal study on infants from birth to 36 months of age from Peru and Tanzania showed plasma tryptophan concentrations were positively associated with linear growth (17). Considering the differences in milk composition and gastric digestion rates between milk from different ruminant species, ruminant milk may influence plasma tryptophan levels in infants, which in turn, could have an impact on their overall growth and development. However, studies have yet to address whether the consumption of milk from different ruminant species in early postnatal life can modulate the appearance of metabolites in the peripheral circulation.

Based on the existing evidence, it was hypothesised that bovine, caprine or ovine milk with different nutrient compositions and gastric digestion rates would lead to a varied abundance of host and microbial metabolites appearing in the peripheral blood circulation. Therefore, the aim was to determine the effects of bovine, caprine, or ovine whole milk treatments on the circulatory blood plasma metabolome in pigs as a model of human infants. The samples were collected as part of a study that focused on structural gastric changes in bovine and non-bovine whole milk in early postnatal life (11). Liquid chromatography-mass spectrometry (LC-MS) metabolomics was used to profile all the metabolites (polar and non-polar) detected in blood plasma. In addition, LC-MS lipidomics was used to profile all the lipids detected in milk. Finally, high-performance liquid chromatography (HPLC) was used to quantify the concentration of amino acids in milk and blood plasma.

## 2. Materials and methods

### 2.1. Milk nutrient analysis

Three batches of milk samples collected during the study to feed the pigs were stored at −20°C before being freeze-dried and ground. The freeze-dried raw whole milk batches were analysed for standard amino acids using AOAC method 994.12, including HCl acid hydrolysis, followed by reversed-phase high-performance liquid chromatography (RP-HPLC). Cysteine and methionine were analysed using AOAC method 994.12, including performic acid oxidation. Tryptophan was analysed using alkaline hydrolysis, followed by RP-HPLC (18, 19). Freeze-dried milk samples were reconstituted with Milli-Q^®^ water for lipid analyses using LC-MS lipidomics.

### 2.2. Animal study

The protocol used for the animal study was approved by the Massey University Animal Ethics Committee (MUAEC protocol 18/97) and described in detail elsewhere (11). Twenty-four male pigs [postnatal day (PND) 7, mean body weight (BW) on arrival 3 kg (1.9–3.5)] were obtained from a local commercial farm (Aorere Farms Partnership, Whanganui, New Zealand). These animals were housed in purpose-built plastic metabolism crates (700 × 450 × 500 mm) in a room with a 16 h light-8 h dark cycle and a static temperature of 28 ± 2°C. The crates of these animals were enriched with toys, which were replaced and cleaned daily. Pigs were allowed to socialise every day under supervision for an hour.

Upon arrival, pigs were randomly assigned to three diet groups (bovine, caprine, or ovine milk) such that there were 8 pigs per milk treatment. From birth to PND 6 (the period before the experimental day), these pigs consumed *ad libitum* sow’s milk. The experimental period comprises PND 7 to PND 21, where pigs were bottle-fed one of the milk treatments. From PND 7 to PND 18, pigs were bottle-fed either bovine, caprine, or ovine reconstituted whole milk powder diets (purchased from Davis Food Ingredients, Dairy Goat Co-operative, and Spring Sheep Milk Co., respectively), including vitamin and mineral supplements (purchased from Nutritech International Ltd.). From PND 19, the pigs were fed either bovine, caprine or ovine fresh whole milk obtained under chilled conditions from the Massey University No. 4 Dairy Farm (Palmerston North, New Zealand), Dairy Goat Co-operative (Hamilton, New Zealand) and Phoenix Goats (Palmerston North, New Zealand), and Neer Enterprises Limited (Carterton, New Zealand), respectively. The fresh milk was provided for 3 days only due to a limited supply of fresh ovine and caprine milk.

The pigs received iso-caloric and iso-volumetric amounts of each diet on a BW basis (345 g of liquid meal per kg of BW per day) from PND 7 to PND 13, which was considered the acclimatisation period for the pigs to learn to drink from the bottle with a rubber teat. The diets were balanced for protein content from PND 14 to PND 21 (2 g per kg BW). From PND 14 to PND 18 (reconstituted powder), the pigs received equal amounts of protein (2 g of protein per kg of BW in every single meal) and equal volumes of diet (345 g of liquid meal per kg of BW per day). From PND 19 (fresh milk), the pigs received their respective milk volumes based on equal amounts of protein per kg of BW. Balancing the protein intake between groups allowed the investigation of the effects of structural changes in ruminants’ milk on raw whole milk gastric digestion, which was the study’s primary objective (11). On the last experimental day, pigs of PND 21 were euthanised at 210 min post-feeding to allow time for nutrient absorption. A diagram illustrating the study timeline is provided as Supplementary Figure S1.

### 2.3. Blood plasma sampling

The pigs were anaesthetised using Zoletil 100 (50 mg/mL each of zolazepam and tiletamine, Zoetis Inc., Parsippany-Troy Hills, NJ, United States) reconstituted with 2.5 mL each of ketamine and xylazine (both 100 mg/mL). Blood samples were drawn from the left ventricle and were collected in evacuated blood collection tubes containing EDTA (BD Vacutainer^®^; Franklin Lakes, NJ, United States). Immediately after the blood collection, the blood sample was centrifuged at 4,500 rpm for 10 min at 4°C, and the plasma was removed and stored at −80°C until required.

### 2.4. Plasma amino acid analysis

The concentration of amino acids in plasma was quantified (AgResearch Analytical Laboratory, Palmerston North, New Zealand) using the Pico-Tag method (20) as described by Milan et al. (21). Briefly, 500 μL of each plasma sample was used for sample preparation, followed by HPLC analysis using the Pico-Tag C18 column 60 Å, 4 μm, 3.9 mm × 300 mm (Waters Corporation, MA, United States).

### 2.5. Metabolomic analysis

#### 2.5.1. Chemicals

All the chemicals and solvents used were LC-MS grade unless specified. Chloroform (analytical grade), methanol, acetonitrile, isopropanol, and formic acid were purchased from Thermo Fisher Scientific (Waltham, MA, United States). Milli-Q^®^ ultrapure water was purchased from Merck Millipore (Bedford, MA, United States). Ammonium formate (HPLC grade) and internal standards (d_5_-tryptophan, d_10_-leucine, d_2_-tyrosine, and d_7_-alanine) used in extraction solvent were purchased from Sigma-Aldrich (St. Louis, MO, United States). SPLASH^®^ lipidomix^®^ mass spectrometry standard was obtained from Avanti^®^ (Alabaster, AL, United States), which included all of the major lipid classes [15:0–18:1(d7) PC, 15:0–18:1(d7) PE, 15:0–18:1(d7) PS, 15:0–18:1(d7) PG, 15:0–18:1(d7) PI, 15:0–18:1(d7) PA, 18:1(d7) LPC, 18:1(d7) LPE, 18:1(d7) Chol Ester,18:1(d7) MG, 15:0–18:1(d7) DG, 15:0–18:1(d7)-15:0 TG, 18:1(d9) SM, Cholesterol (d7)].

#### 2.5.2. Sample preparation

Plasma samples were thawed overnight at 4°C and vortexed. Extraction solvent of 800 μL containing chloroform: methanol (1:1 v/v containing internal standards d_5_-tryptophan, d_10_-leucine, d_2_-tyrosine, and d_7_-alanine), precooled at −20°C was added to each 2 mL microcentrifuge tubes containing 100 μL of plasma. The mixture was vortexed for 30 s and then incubated for 60 min at −20°C. Then 400 μL of Milli-Q^®^ water was added to each sample, vortexed for 30 s and centrifuged for 10 min at 11,000 rpm at 4°C. Subsequently, 200 μL aliquots of the supernatant and 250 μL of the bottom layer were transferred into new 2 mL microcentrifuge tubes for polar and non-polar metabolites analyses. The pooled polar quality control samples were prepared by combining 100 μL of the supernatant from each sample into a new tube, vortexed for 30 s and then aliquoted into multiple microcentrifuge tubes. Similar procedures were followed for non-polar quality control samples, except the aliquots of 80 μL were taken from the bottom layer. Blank samples were prepared using the above procedures, except the samples were replaced with 100 μL Milli-Q^®^ water. All samples and blanks were dried under a stream of nitrogen at room temperature and stored at −80°C.

On the day of LC-MS analysis, the dried extracts were reconstituted in 300 μL of acetonitrile: water (1:1 v/v) containing formic acid (0.1%) for polar metabolites and 800 μL of chloroform: methanol (2:1 v/v) for non-polar metabolites. The reconstituted polar metabolite mixture was vortexed for 15 s, then centrifuged for 10 min at 11,000 rpm at 4°C. Aliquots of 100 μL of polar extract and 200 μL of lipid extracts were transferred into a vial containing a volume insert. Then, 7 μL of SPLASH^®^ lipidomix^®^ was added to the insert containing the lipid extract only. The inserts containing the metabolite extracts were stored at 4°C for immediate metabolite analysis.

#### 2.5.3. Liquid chromatography-mass spectrometry analysis

Metabolites were analysed using a LC-MS-9030 mass spectrometer coupled with a Nexera-x2 ultra-performance liquid chromatography system (Shimadzu, Kyoto, Kyoto, Japan) as described by Abshirini et al. (22). Briefly, chromatographic separations of polar and non-polar metabolites were conducted by injecting 5 μL of samples onto Accucore^™^ HILIC column, 2.1 mm × 100 mm, 2.6 μm particle size (Thermo Fisher Scientific, Waltham, MA, United States), and 2 μL of samples onto the CSH-C18 column, 2.1 × 100 mm, 1.7 μm particle size (Waters, Milford, MA, United States), respectively. The mobile phases used for chromatographic separations were 10 mM ammonium formate in water (solvent A) and 0.1% of formic acid in acetonitrile (solvent B) for polar metabolites and 10 mM of ammonium formate in water/acetonitrile/isopropanol (5:3:2 v/v) (solvent A) and 10 mM of ammonium formate in water/acetonitrile/isopropanol (1:9:90 v/v) (solvent B) for non-polar metabolites. The mass spectral detection for polar metabolites was performed in positive and negative ionisation modes. In contrast, for non-polar metabolites, mass spectral detection was only performed in positive ionisation mode, as it captures most lipids.

#### 2.5.4. Data processing

Raw data files were converted to centroid mzML format using the Shimadzu file converter and were uploaded to MS-DIAL software (version 4.48) (23) for subsequent data-processing steps, including peak detection, gap-filling, alignment, and noise removal. The processing parameters were kept at default except for minimum peak height and retention time tolerance. For polar metabolite data analysed in positive and negative ionisation modes, minimum peak heights were 1,000 and 1,500, respectively, and retention time tolerances were 0.15 min and 0.3 min, respectively. For non-polar metabolite data analysed in positive ionisation mode, minimum peak height was 1,000 and retention time tolerance was 0.15 min. MS data was acquired in data independent acquisition mode to enable MS/MS spectral reconstruction. Data independent acquisition MS/MS spectra were used for aligned peak identification. MS/MS public library containing 13,303 unique compounds (23) and the built-in lipid library containing 257,000 *in silico* generated MS/MS lipid fragmentation spectra were used for polar and non-polar metabolite feature identification, respectively. Afterwards, data normalisation was conducted using the quality control samples (LOESS normalisation) and the features with a quality control coefficient of variation >30% were removed. Identified missing values were treated with the k-nearest neighbour method using Metaboanalyst (version 5.0) (24). The human metabolome database (25) and Metlin (26) were used to search unknown polar metabolites features based on their m/z with a mass error value of less than 15 ppm.

Similar to the procedures applied to plasma non-polar sample preparations, LC-MS run and data processing were followed for milk lipidomic analysis except for samples volume, i.e., 200 μL each of extracted sample was used for milk lipidomic analysis.

### 2.6. Statistical analysis

The concentration of amino acids in plasma samples and three batches of each milk treatment were analysed using a one-way analysis of variance (ANOVA) of the rstatix R (version 4.02) package. Amino acid concentrations with a false discovery rate (FDR) < 0.05 between milk treatments were considered significantly different. The Fisher’s least significant difference test was used for *post hoc* analysis on FDR-adjusted ANOVA, performed using the agricolae package for R.

Milk lipids’ relative intensities data were expressed as relative percentage (%) for calculating the lipid intake in the last meal. The percentage of each lipid was calculated within each lipid class. Lipids within the same class, e.g., TGs, ionise at a similar rate, whereas lipids belonging to different classes ionise differently in the electrospray source; hence, the lipids were not expressed as a percentage of total lipids but as a percentage within the lipid class.

Multivariate statistical analyses, including principal component analysis (PCA) and partial least squares discriminant analysis (PLS-DA), were conducted to investigate differences in polar and non-polar metabolite profiles in plasma samples in response to different milk treatments using SIMCA (version 16). Validation of the PLS-DA model was performed using the predictive ability of the model (Q^2^) and analysis of variance of cross-validated residuals (CV-ANOVA), i.e., *Q*^2^ value approximately >0.5 and CV-ANOVA *p*-value < 0.05, were considered as a good model for multivariate data. Permutation tests involving 100 permutations were used to check the robustness of the model. Features with a variable important for projection (VIP) score > 1 identified using the PLS-DA model were used for metabolites selection. One-way ANOVA was conducted on selected metabolites using the Metaboanalyst platform (version 5.0) (24). Metabolites with an FDR < 0.05 were considered significant. On the significant metabolites, as identified by FDR-adjusted ANOVA, a pair-wise plasma metabolite relative intensity fold change (FC) was calculated between milk treatment groups (ovine vs. caprine, ovine vs. bovine, and caprine vs. bovine). The pairwise FC satisfying the criteria of Log_2_FC > ±1 (equivalent to FC > 2) and FDR < 0.05 (identified using *t*-tests) was considered significant. Metaboanalyst (version 5.0) was used for heatmap visualisation and hierarchical clustering was performed using the Ward’s method.

The association between milk nutrient intake (amino acids and lipids) in the last meal and plasma metabolites were assessed using Spearman correlations. Correlations with *p* < 0.05 and rho > ±0.5 were considered significant and visualised using the corrplot package in R. Last meal intake was used for correlation analysis as the nutrient appearance in the peripheral blood is expected to be due to the last meal after 18 h of fasting.

## 3. Results

### 3.1. Milk amino acid profiles

Ovine milk had significantly higher (FDR < 0.05) concentrations of all the amino acids and total amino acid (TAA), essential amino acid (EAA), branched chain amino acid (BCAA), and large neutral amino acid (LNAA) compared to bovine milk and caprine milk (Table 1). Bovine milk had significantly higher (FDR < 0.05) concentrations of all amino acids and TAA, EAA, BCAA, and LNAA than caprine milk, except arginine, glutamic acid, isoleucine, and valine (FDR > 0.05).

**Table 1:** Amino acid composition of bovine, caprine, and ovine raw whole milk, and amino acid provided in the last meal*.

Amino acid	Raw whole milk (mg/mL)				Intake (mg)			
	Bovine	Caprine	Ovine	*F*-value	Bovine	Caprine	Ovine	*F*-value
Alanine	1.10 ± 0.02^b^	0.90 ± 0.08^c^	2.13 ± 0.06^a^	386.58	288.96 ± 40.35	289.5 ± 33.24	336.52 ± 37.13	4.35
Arginine	1.16 ± 0.07^b^	0.87 ± 0.09^c^	2.04 ± 0.13^a^	113.14	303.81 ± 42.43	278.42 ± 31.96	322.42 ± 35.58	2.86
Aspartic acid	2.54 ± 0.13^b^	2.15 ± 0.07^c^	4.56 ± 0.04^a^	605.53	667.75 ± 93.25	692.58 ± 79.52	720.64 ± 79.52	0.79
Cysteine	0.21 ± 0.01^b^	0.21 ± 0.02^b^	0.39 ± 0.03^a^	86.22	55.94 ± 7.81	65.5 ± 7.52	62.23 ± 6.87	3.44
Glutamic acid	6.88 ± 0.13^b^	5.93 ± 0.16^c^	11.47 ± 0.45^a^	318.22	1805.17 ± 252.09	1907.77 ± 219.03	1813.03 ± 200.05	0.52
Glycine	0.70 ± 0.01^b^	0.55 ± 0.03^c^	1.21 ± 0.02^a^	827.37	184.58 ± 25.78	178.57 ± 20.50	191.48 ± 21.13	0.65
Histidine	0.85 ± 0.03^b^	0.75 ± 0.03^c^	1.48 ± 0.04^a^	460.12	224.28 ± 31.32	241.86 ± 27.77	233.88 ± 25.81	0.77
Isoleucine	1.69 ± 0.07^b^	1.39 ± 0.07^c^	2.74 ± 0.02^a^	450.66	443.37 ± 61.91	448.37 ± 51.48	432.95 ± 47.77	0.17
Leucine	3.20 ± 0.15^b^	2.75 ± 0.05^c^	5.53 ± 0.22^a^	265.38	839.08 ± 117.18	885.55 ± 101.67	874.36 ± 96.48	0.42
Lysine	2.72 ± 0.06^b^	2.36 ± 0.04^c^	4.65 ± 0.23^a^	239.88	712.68 ± 99.52	758.6 ± 87.10	734.54 ± 81.05	0.53
Methionine	0.89 ± 0.03^b^	0.74 ± 0.02^c^	1.60 ± 0.06^a^	345.51	232.32 ± 32.44	236.76 ± 27.18	252.43 ± 27.85	1.04
Phenylalanine	1.57 ± 0.07^b^	1.40 ± 0.02^c^	2.61 ± 0.06^a^	434.11	410.83 ± 57.37	450.45 ± 51.72	412.3 ± 45.50	1.51
Proline	3.31 ± 0.09^b^	3.11 ± 0.14^b^	5.91 ± 0.18^a^	367.53	869.33 ± 121.40	999.49 ± 114.75	933.48 ± 103.00	2.64
Serine	1.74 ± 0.06^b^	1.43 ± 0.05^c^	2.82 ± 0.12^a^	227.23	456.41 ± 63.74	459.76 ± 52.79	445.45 ± 49.15	0.15
Threonine	1.54 ± 0.10^b^	1.53 ± 0.08^b^	2.38 ± 0.09^a^	83.82	403.85 ± 56.40^b^	492.9 ± 56.59^a^	375.54 ± 41.44^b^	11.11
Tryptophan	0.47 ± 0.01^b^	0.40 ± 0.02^c^	0.83 ± 0.01^a^	803.02	122.84 ± 17.15	129.93 ± 14.92	131.61 ± 14.52	0.72
Tyrosine	1.68 ± 0.06^b^	1.16 ± 0.06^c^	2.67 ± 0.09^a^	347.59	440.46 ± 61.51	371.69 ± 42.67	421.63 ± 46.52	3.91
Valine	2.17 ± 0.09^b^	2.09 ± 0.08^b^	3.69 ± 0.12^a^	256.06	568.68 ± 79.42	671.03 ± 77.04	583.54 ± 64.39	4.48
TAA	34.41 ± 1.02^b^	29.71 ± 0.85^c^	58.71 ± 1.64^a^	489.45	9030.33 ± 1261.06	9558.71 ± 1097.45	9278.05 ± 1023.73	0.44
EAA	15.08 ± 0.57^b^	13.41 ± 0.29^c^	25.51 ± 0.75^a^	396.89	3957.92 ± 552.71	4315.44 ± 495.46	4031.17 ± 444.80	1.14
BCAA	7.05 ± 0.31^b^	6.23 ± 0.20^c^	11.96 ± 0.35^a^	328.96	1851.13 ± 258.51	2004.94 ± 230.19	1890.85 ± 208.64	0.94
LNAA	14.04 ± 0.57^b^	12.21 ± 0.26^c^	23.53 ± 0.60^a^	439.73	3685.7 ± 514.70	3928.52 ± 451.04	3718.26 ± 410.27	0.65

*Data analysed via one-way ANOVA with *post hoc* Fisher’s least significant difference test. Values are represented as mean ± standard deviation. ^a-c^Values in the same row without a common superscript are different (FDR < 0.05) from each other. TAA, total amino acid; EAA, essential amino acid; BCAA, branched-chain amino acid; LNAA, large neutral amino acids.

For the last feed, protein contents (2 g of protein per kg BW) (Supplementary Table S1) were matched between milk treatments, resulting in similar amino acid intakes between milk treatments (Table 1). Only threonine intake was different between milk, i.e., pigs fed caprine milk consumed more threonine than bovine and ovine milk groups.

### 3.2. Milk lipid profiles

Four hundred and ninety-five features were detected during the initial lipidomics data analysis. After filtration and removal of background noise, 88 features were identified and used for subsequent statistical analyses.

**Figure 1:**
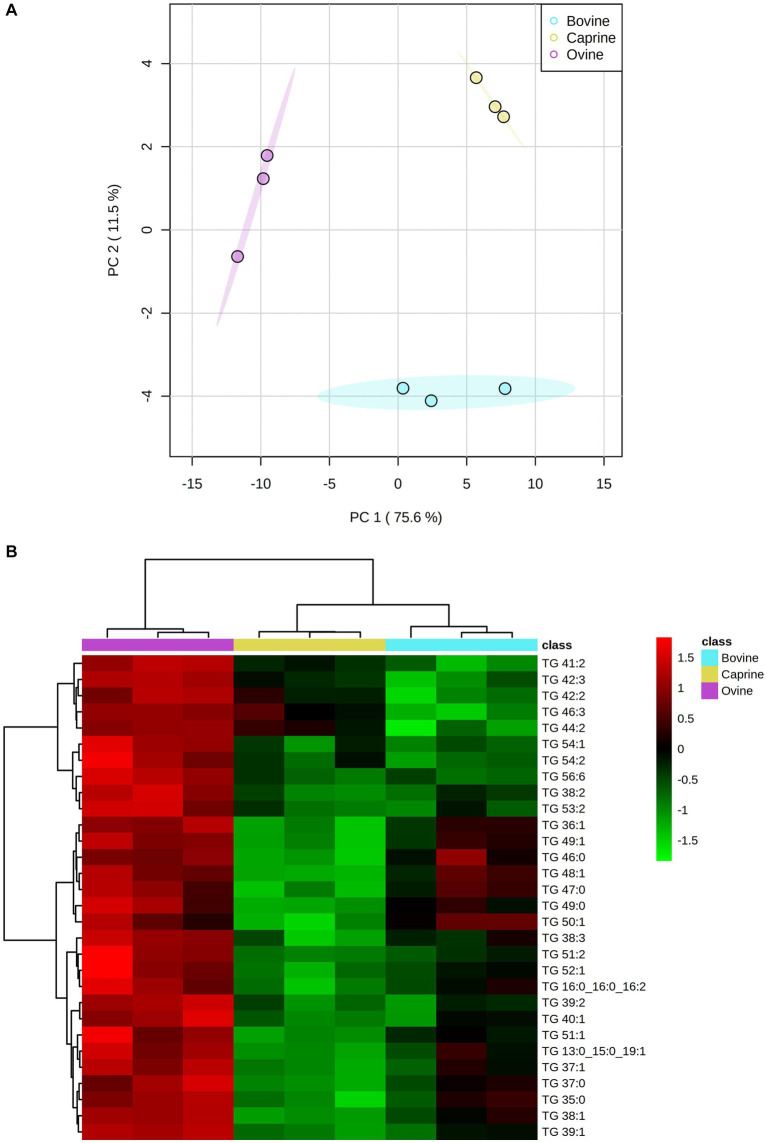
**(A)** Score plot of principal component analysis showing lipid relative intensity differences between bovine, caprine and ovine milk. The first two principal components are plotted. Percentages of variation explained by each principal components (PC) are indicated along the axes. **(B)** Heatmap showing hierarchical clustering [method, Ward] of top 30 significantly different lipids between bovine (aqua), caprine (yellow), and ovine (purple) milk samples. Heatmap colour indicates normalised (Z score) peak intensity of lipids. The intensity of the red colour denotes the number of standard deviations above the mean (higher relative intensity), and the intensity of green colour denotes the number of standard deviations below the mean (lower relative intensity). TG, triglyceride.

The PCA score plot showed a clear separation between milk treatments (Figure 1A). A hierarchical cluster analysis confirmed the lipids grouping by the PCA model (Figure 1B). One-way ANOVA analysis showed that 81 out of 88 lipids identified differed between milk groups (Supplementary Table S2). These selected lipids were used further for pair-wise comparison between milk groups, as shown in Supplementary Table S2. The relative intensity FC of 22 triglycerides (TG) in ovine milk was significantly higher (log_2_FC > ±1 and FDR < 0.05) than in bovine milk (Supplementary Table S2). The relative intensity FC of 36 TG in ovine milk was significantly higher than in caprine milk. Relative intensity FC between bovine and caprine milk lipids was similar.

As per the study design, pigs received different volumes of each milk type to compensate for the balanced protein intake, which also resulted in similar total fat intake between the milk treatments (Supplementary Table S1). However, there were differences in intake of specific lipids between milk (Table 2). For example, pigs fed ovine milk generally had a higher TG intake than caprine or bovine milk groups. Similarly, pigs fed with caprine milk generally had a lower intake of TG than those fed ovine or bovine milk.

**Table 2:** Lipid composition of bovine, caprine, and ovine raw whole milk, and lipids provided in the last meal.

Lipid*	Raw whole milk (%)			Intake** (mg)^+^			
	Bovine	Caprine	Ovine	Bovine	Caprine	Ovine	*F*-value
TG 8:0_12:0_14:0	3.00	2.51	3.05	319.2 ± 44.62^a^	261.24 ± 29.99^b^	303.63 ± 33.48^a^	5.38
TG 9:0_9:0_16:0	1.66	1.39	1.67	176.97 ± 24.74^a^	144.96 ± 16.64^b^	166.48 ± 18.36^a^	5.22
TG 10:0_14:0_16:0	2.34	2.86	2.52	248.83 ± 34.78^b^	297.35 ± 34.13^a^	251.75 ± 27.76^b^	5.65
TG 12:0_12:0_16:0	1.26	1.47	1.40	134.37 ± 18.78	152.41 ± 17.49	139.54 ± 15.39	2.31
TG 12:0_14:0_16:0	2.18	2.74	2.31	232.66 ± 32.52^b^	284.92 ± 32.70^a^	230.19 ± 25.38^b^	8.27
TG 13:0_15:0_19:1	0.62	0.51	0.65	65.93 ± 9.22^a^	52.90 ± 6.07^b^	65.23 ± 7.17^a^	7.32
TG 14:0_14:0_14:0	0.90	1.21	0.96	95.43 ± 13.34^b^	125.39 ± 14.39^a^	95.26 ± 10.5^b^	14.57
TG 14:0_16:0_18:2	1.99	1.71	1.71	211.55 ± 29.57^a^	177.96 ± 20.43^b^	170.28 ± 18.78^b^	7.03
TG 14:0_18:1_18:1	4.32	3.94	3.63	460.28 ± 64.34^a^	409.15 ± 46.96^ab^	362.04 ± 39.92^b^	7.3
TG 16:0_16:0_16:2	0.46	0.43	0.43	49.12 ± 6.85	44.68 ± 5.13	42.92 ± 4.73	2.46
TG 16:0_16:0_18:2	0.60	0.55	0.50	63.95 ± 8.94^a^	57.22 ± 6.57^ab^	50.18 ± 5.53^b^	7.4
TG 16:0_16:0_20:3	0.36	0.42	0.41	38.63 ± 5.40	43.36 ± 4.97	40.86 ± 4.50	1.81
TG 16:0_18:1_18:2	2.17	2.59	2.73	230.74 ± 32.26^b^	268.71 ± 30.84^a^	271.8 ± 29.97^a^	4.34
TG 26:0	0.06	0.07	0.15	6.10 ± 0.85^b^	7.07 ± 0.81^b^	15.16 ± 1.67^a^	142.06
TG 28:0	0.16	0.15	0.33	32.93 ± 2.37^a^	15.85 ± 1.82^b^	32.43 ± 3.58^a^	95.23
TG 30:0	0.21	0.31	0.38	38.63 ± 3.74^a^	32.63 ± 3.74^b^	37.70 ± 4.16^a^	35.61
TG 32:0	0.43	0.35	0.50	45.34 ± 6.34^a^	36.11 ± 4.15^b^	49.36 ± 5.44^a^	12.74
TG 32:1	0.11	0.20	0.16	11.53 ± 1.61^c^	20.45 ± 2.35^a^	15.62 ± 1.72^b^	43.2
TG 33:0	0.14	0.09	0.16	14.73 ± 2.06^a^	9.87 ± 1.13^b^	16.15 ± 1.78^a^	29.92
TG 35:0	0.61	0.45	0.63	64.55 ± 9.02^a^	47.02 ± 5.40^b^	63.07 ± 6.95^a^	14.28
TG 36:0	2.84	2.28	2.29	303.08 ± 42.37^a^	236.99 ± 27.21^b^	228.55 ± 25.20^b^	12.61
TG 36:1	2.12	1.66	1.96	225.35 ± 31.52^a^	172.84 ± 19.84^b^	195.53 ± 21.56^b^	8.99
TG 37:0	0.64	0.53	0.67	68.57 ± 9.59^a^	54.82 ± 6.29^b^	66.91 ± 7.38^a^	7.27
TG 37:1	0.36	0.29	0.45	38.75 ± 5.42^b^	30.21 ± 3.47^c^	44.84 ± 4.95^a^	19.72
TG 38:0	3.59	3.30	3.27	382.12 ± 53.42	343.03 ± 39.37	326.22 ± 35.97	3.46
TG 38:1	2.05	1.82	1.96	218.02 ± 30.48	188.68 ± 21.66	195.39 ± 21.55	3.05
TG 38:2	0.89	0.92	1.21	94.36 ± 13.19^b^	95.85 ± 11^b^	120.48 ± 13.28^a^	10.96
TG 38:3	0.11	0.06	0.22	11.61 ± 1.62^b^	6.62 ± 0.76^c^	21.53 ± 2.37^a^	156.56
TG 39:0	0.37	0.39	0.49	39.94 ± 5.58^b^	40.18 ± 4.61^b^	48.68 ± 5.37^a^	7.32
TG 39:1	0.38	0.36	0.48	40.78 ± 5.7^b^	37.33 ± 4.28^b^	47.96 ± 5.29^a^	8.96
TG 39:2	0.08	0.08	0.19	8.74 ± 1.22^b^	8.50 ± 0.97^b^	18.85 ± 2.08^a^	123.83
TG 40:1	2.78	2.87	3.02	296.12 ± 41.38	298.65 ± 34.28	301.46 ± 33.24	0.04
TG 41:0	0.32	0.38	0.43	34.51 ± 4.82^b^	39.85 ± 4.57^a^	42.54 ± 4.69^a^	6.05
TG 41:1	0.21	0.28	0.29	22.68 ± 3.17^b^	29.59 ± 3.41^a^	29 ± 3.20^a^	11.07
TG 41:2	0.03	0.07	0.14	3.69 ± 0.52^c^	7.53 ± 0.86^b^	13.63 ± 1.52^a^	184.31
TG 42:1	1.65	2.35	2.04	175.85 ± 24.58^c^	244.39 ± 28.05^a^	203.78 ± 22.47^b^	15.04
TG 42:2	0.71	1.14	0.92	75.15 ± 10.50^c^	118.23 ± 13.57^a^	91.63 ± 10.10^b^	28.59
TG 42:3	0.23	0.37	0.39	24.49 ± 3.42^b^	38.34 ± 4.40^a^	39.2 ± 4.32^a^	32.91
TG 43:0	0.36	0.39	0.43	38.74 ± 5.41	40.93 ± 4.71	43.07 ± 4.75	1.52
TG 43:1	0.23	0.26	0.39	24.14 ± 3.37^b^	27.01 ± 3.11^b^	38.67 ± 4.26^a^	36.31
TG 43:2	0.05	0.05	0.10	4.93 ± 0.69^b^	5.02 ± 0.58^b^	9.55 ± 1.05^a^	87.51
TG 44:0	2.53	2.66	2.22	269.34 ± 37.65^a^	276.79 ± 31.77^a^	221.71 ± 24.45^b^	7.09
TG 44:1	2.01	3.10	2.49	213.73 ± 29.88^c^	321.8 ± 36.94^a^	247.8 ± 27.32^b^	24.4
TG 44:2	0.62	1.21	0.96	65.97 ± 9.22^c^	125.51 ± 14.41^a^	95.71 ± 10.55^b^	52.64
TG 44:3	0.20	0.34	0.34	21.46 ± 3.01^b^	35.41 ± 4.07^a^	33.44 ± 3.69^a^	34.97
TG 45:0	0.60	0.45	0.53	63.40 ± 8.86^a^	46.48 ± 5.34^b^	52.47 ± 5.79^b^	12.58
TG 45:1	0.32	0.33	0.37	33.99 ± 4.75	34.61 ± 3.97	36.93 ± 4.07	1.05
TG 46:0	3.21	2.55	2.42	341.84 ± 47.79^a^	265.09 ± 30.43^b^	241.07 ± 26.58^b^	16.98
TG 46:1	3.24	3.20	2.81	345.39 ± 48.28^a^	332.3 ± 38.14^a^	279.74 ± 30.85^b^	6.11
TG 46:2	0.94	1.50	1.16	100.07 ± 13.99^b^	155.45 ± 17.85^a^	115.23 ± 12.71^b^	29.09
TG 46:3	0.24	0.51	0.41	25.13 ± 3.51^c^	52.63 ± 6.04^a^	40.98 ± 4.52^b^	66.02
TG 47:0	0.78	0.55	0.65	82.94 ± 11.59^a^	57.04 ± 6.55^b^	65.22 ± 7.19^b^	18.37
TG 48:0	2.75	2.17	2.00	13.15 ± 1.20^a^	10.66 ± 1.22^b^	13.05 ± 1.44^a^	19.83
TG 48:1	6.11	4.71	4.89	293.15 ± 40.98^a^	225.13 ± 25.84^b^	199.57 ± 22.01^b^	14.71
TG 48:3	0.41	0.37	0.40	43.66 ± 6.11	38.19 ± 4.38	40.04 ± 4.41	2.45
TG 49:0	0.58	0.47	0.52	61.55 ± 8.61^a^	48.49 ± 5.57^b^	52.22 ± 5.76^b^	7.85
TG 49:1	1.32	0.95	1.28	140.15 ± 19.59^a^	98.88 ± 11.35^b^	127.37 ± 14.05^a^	15.09
TG 49:2	0.43	0.36	0.45	45.88 ± 6.41^a^	37.77 ± 4.34^b^	44.4 ± 4.90^a^	5.34
TG 49:3	0.06	0.07	0.13	6.32 ± 0.89^c^	7.73 ± 0.89^b^	12.91 ± 1.42^a^	80.32
TG 50:0	1.92	1.80	1.39	204.31 ± 28.56^a^	186.66 ± 21.43^a^	138.8 ± 15.31^b^	18.27
TG 50:1	7.69	6.01	5.48	819.04 ± 114.49^a^	624.69 ± 71.71^b^	546.23 ± 60.23^b^	21.64
TG 50:3	1.06	0.96	1.17	113.23 ± 15.83^ab^	99.39 ± 11.41^b^	117.14 ± 12.92^a^	3.81
TG 50:4	0.20	0.18	0.25	21.29 ± 2.98^b^	18.93 ± 2.17^b^	24.97 ± 2.75^a^	10.5
TG 51:0	0.29	0.27	0.31	30.58 ± 4.27	27.62 ± 3.17	31.33 ± 3.46	2.29
TG 51:1	0.99	0.89	1.01	105.82 ± 14.79	92.03 ± 10.57	101.19 ± 11.16	2.6
TG 51:2	0.74	0.73	0.94	78.72 ± 11.01^b^	75.49 ± 8.66^b^	93.93 ± 10.36^a^	7.67
TG 51:3	0.20	0.22	0.31	21.46 ± 3.01^b^	23.29 ± 2.67^b^	30.94 ± 3.41^a^	21.84
TG 52:1	3.57	3.65	3.23	380.39 ± 53.17^a^	378.97 ± 43.5^a^	322.37 ± 35.55^b^	4.39
TG 52:2	5.63	5.79	5.60	599.86 ± 83.86	602.27 ± 69.13	557.89 ± 61.52	0.96
TG 52:4	0.67	0.68	0.99	71.02 ± 9.93^b^	70.51 ± 8.1^b^	98.43 ± 10.86^a^	21.72
TG 52:5	0.11	0.07	0.20	11.35 ± 1.59^b^	7.11 ± 0.81^c^	19.85 ± 2.19^a^	129.06
TG 53:2	0.41	0.47	0.48	43.30 ± 6.05	48.46 ± 5.56	47.48 ± 5.24	1.9
TG 53:3	0.18	0.21	0.29	19.44 ± 2.72^b^	21.99 ± 2.53^b^	28.69 ± 3.16^a^	23.07
TG 53:4	0.04	0.06	0.10	4.41 ± 0.62^c^	6.24 ± 0.72^b^	9.77 ± 1.08^a^	86.62
TG 54:0	0.12	0.14	0.14	12.76 ± 1.78	14.38 ± 1.65	14.06 ± 1.55	2.12
TG 54:1	0.64	0.80	0.81	68.13 ± 9.52^b^	83.16 ± 9.54^a^	81.17 ± 8.95^a^	6.11
TG 54:2	1.47	2.02	2.00	156.79 ± 21.92^b^	209.86 ± 24.09^a^	199.73 ± 22.02^a^	12.33
TG 54:3	1.69	2.51	2.31	179.91 ± 25.15^c^	261.31 ± 29.99^a^	230.14 ± 25.38^b^	18.6
TG 54:4	0.81	1.24	1.27	86.23 ± 12.05^b^	129.32 ± 14.84^a^	126.53 ± 13.95^a^	24.9
TG 54:5	0.33	0.46	0.52	34.87 ± 4.88^b^	48.31 ± 5.54^a^	51.99 ± 5.73^a^	22.29
TG 56:6	0.03	0.04	0.14	3.45 ± 0.48^b^	4.08 ± 0.47^b^	13.56 ± 1.50^a^	286.14

*Only the lipids whose relative intensities were significant between original milk are represented as a percentage. **Data analysed via one-way ANOVA with *post hoc* Fisher’s least significant difference test and values are represented as mean ± standard deviation. ^a-c^Values in the same row without a common superscript are different (FDR < 0.05) from each other. ^+^Intake is represented as the amount of each TG in the total amount of fat consumed in the last meal. TG, Triglycerides.

### 3.3. Plasma polar metabolites

**Figure 2:**
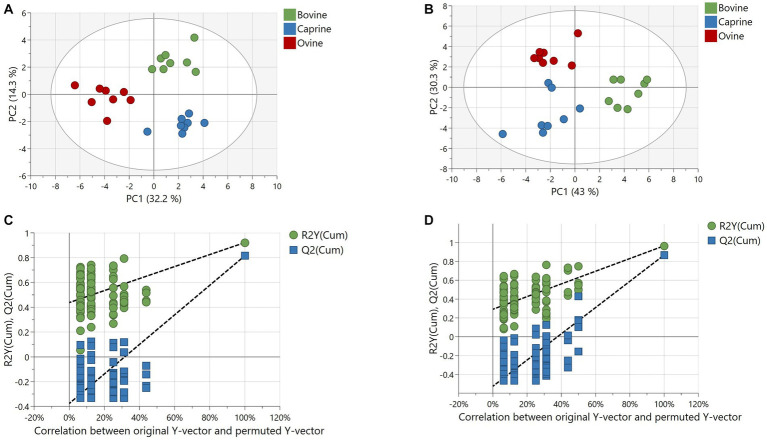
Partial least squares discriminant analysis (PLS-DA) of metabolites relative intensity differences in plasma of pigs fed bovine, caprine, or ovine milk treatment. **(A)** Score plot and **(B)** permutation plot of polar metabolites. **(C)** Score plot and **(D)** permutation plot of non-polar metabolites. Permutation plots involve 100 permutation tests showing no overfitting of the PLS-DA model, confirming the robustness of the model. The criteria for evaluating whether there is overfitting of the PLS-DA model is that Q2 and R2 values of the permutated Y model to the left were lower than the Q2 and R2 value of the original model to the far right. The first two principal components are plotted. Percentages of variation explained by each principal component are indicated along the axes. PC, principal component.

The number of features obtained in positive and negative ionisation modes from the polar analytical stream was 621 and 120, respectively. The positive and negative ionisation data were combined into one dataset after filtration and background noise removal. The resulting 318 features (43 known and 275 unknowns) were used for multivariate analyses. The PCA model could not resolve any separation of polar plasma metabolites between milk treatments (Supplementary Figure S2A). However, a validated PLS-DA model (Q2 = 0.81 and CV-ANOVA *p*-value < 0.05) with a good fit showed a clear and robust separation of polar plasma metabolites between milk treatments (Figures 2A,C).

**Figure 3:**
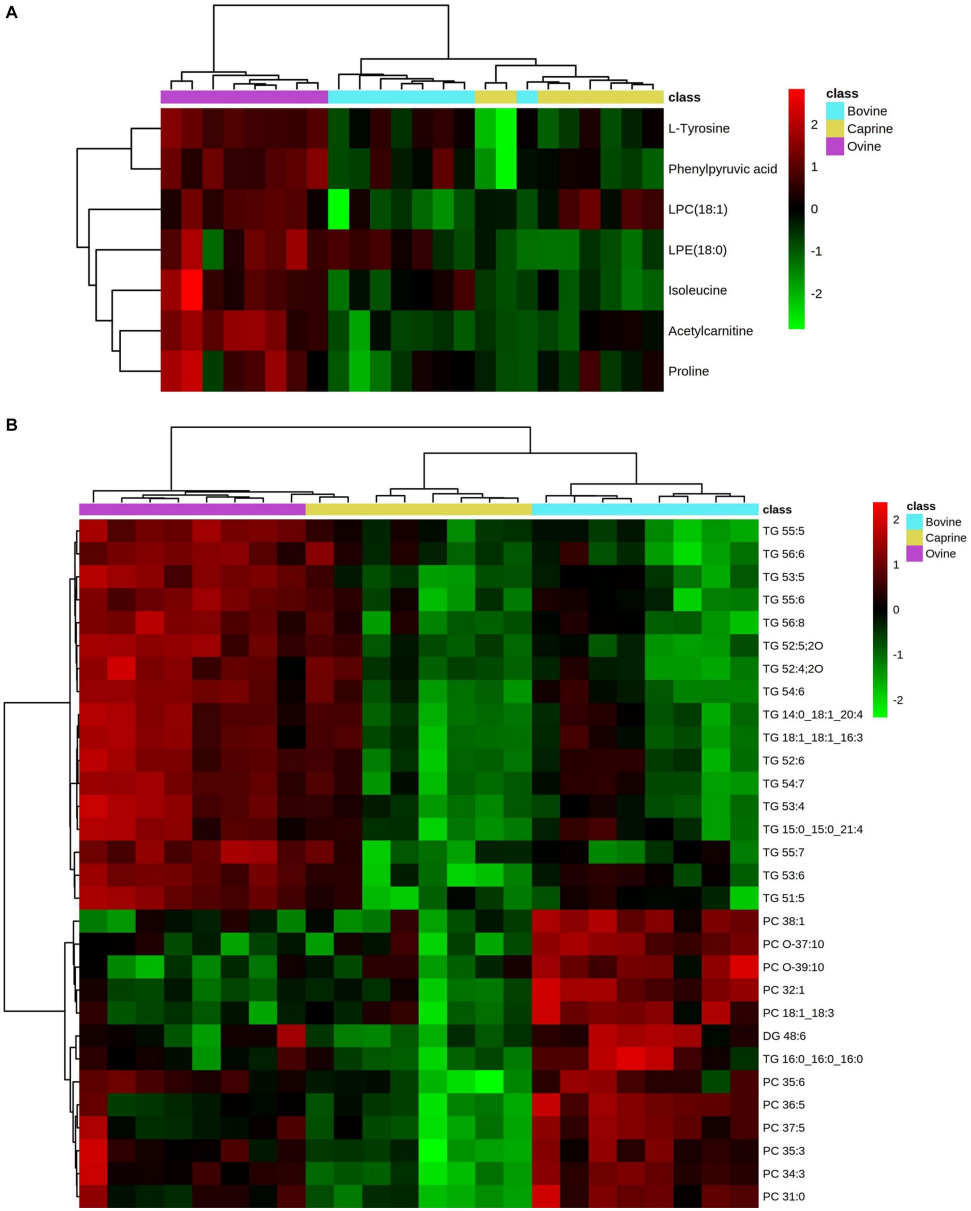
Heatmap showing relative intensities of significantly different. **(A)** polar metabolites and **(B)** lipids (top 30 significant lipids) in plasma of pigs fed bovine, caprine, or ovine milk treatment. Heatmap colour indicates normalised (Z score) peak intensity of metabolites. The intensity of the red colour denotes the number of standard deviations above the mean (higher relative intensity), and the intensity of the green colour denotes the number of standard deviations below the mean (lower relative intensity).

The relative intensity of seven plasma polar metabolites was significantly different (FDR < 0.05) between milk treatments and contributed the most to the separation between milk treatments (VIP > 1) (Table 3). Out of the seven features, three features were unknown during the initial processing, which were putatively identified using human metabolome database search as phenyl pyruvic acid [measured mass (m/z): 165.0533, retention time (min): 8.81], lysophosphatidylcholine (LPC) (18:1) (actual mass: 544.3366, retention time: 5.619), and lysophosphatidylethanolamine (LPE) (18:0) (actual mass: 482.3202, retention time: 5.798). The relative intensity FC of acetylcarnitine and LPC (18:1) in the ovine milk group was significantly higher (log_2_FC > ± 1 & FDR < 0.05) than in the bovine milk group (Table 3). No significant relative intensity FC of metabolites was identified between ovine and caprine milk groups and between caprine and bovine milk groups (Table 3). Overall, the relative intensity of plasma polar metabolites in the ovine milk group was higher compared to the caprine or bovine milk group (Figure 3A).

**Table 3:** Polar metabolites with a significant difference in relative intensities in plasma of pigs fed bovine, caprine, or ovine milk treatment ^+^.

Polar metabolites	VIP	*F*-value	FDR	Log_2_fold change		
				Ovine vs. Bovine	Ovine vs. Caprine	Caprine vs. Bovine
Acetylcarnitine	2.85	37.41	< 0.01	1.05*	0.81	0.24
LysoPC (18:1)	2.64	10.21	0.00	1.14*	0.24	0.89
Isoleucine	2.52	16.18	<0.01	0.36	0.50	−0.14
L-Tyrosine	2.50	13.83	<0.01	0.35	0.70	−0.35
Phenyl pyruvic acid	2.40	9.64	0.01	0.41	0.71	−0.30
LysoPE (18:0)	2.32	5.92	0.03	0.26	0.59	−0.32
Proline	2.18	8.96	0.01	0.40	0.30	0.10

^+^VIP value >1 from the partial least squares discriminant analysis model was used to select the major contributing polar metabolites. One-way ANOVA was used to determine the significant polar metabolites between milk treatments (FDR < 0.05). Only polar metabolites that satisfied multivariate and ANOVA analysis criteria are shown. *Polar metabolites with significant fold change > ±1 (FDR < 0.05 using *t*-test) between treatments. FDR, false discovery rate; VIP, variable importance in projection.

Most plasma polar metabolites identified using LC-MS metabolomics were amino acids. Their concentrations were then quantified using the HPLC method. The plasma concentration of five amino acids, namely asparagine, leucine, lysine, proline, and tyrosine were significantly higher (FDR < 0.05) in the ovine milk treatment group compared to bovine or caprine milk treatment groups (Table 4). In addition, the plasma concentration of taurine and threonine was higher in the caprine milk treatment group compared to the bovine or ovine milk treatment groups (Table 4).

**Table 4:** Amino acid concentrations (μmol/L) in plasma of pigs fed bovine, caprine, or ovine milk treatment*.

Amino acids	Bovine	Caprine	Ovine	FDR	*F*-value
Alanine	522.69 ± 126.84	609.15 ± 128.82	554.6 ± 87.89	0.47	1.14
Arginine	59.59 ± 5.12	56.89 ± 10.19	81.09 ± 29.46	0.08	4.23
Asparagine	68.93 ± 17.11^b^	70.54 ± 7.62^b^	94.03 ± 13.22^a^	0.01	9.02
Aspartic acid	11.64 ± 3.17	9.43 ± 2.59	13.44 ± 4.18	0.18	2.83
Citrulline	124.85 ± 37.21	118.63 ± 19.73	122.90 ± 23.64	0.98	0.10
Cystine	6.66 ± 5.92	11.04 ± 6.53	12.34 ± 10.37	0.47	1.15
Glutamic acid	156.41 ± 40.49	158.1 ± 49.65	160.80 ± 32.36	0.98	0.02
Glutamine	435.42 ± 105.02	485.90 ± 108.00	539.50 ± 117.12	0.29	1.79
Glycine	1214.04 ± 316.78	1385.81 ± 223.85	1077.39 ± 260.22	0.19	2.63
Histidine	48.83 ± 14.23	46.11 ± 8.61	49.95 ± 7.91	0.93	0.29
Hydroxyproline	148.56 ± 33.67	183.58 ± 25.42	184.31 ± 38.98	0.17	3.02
Isoleucine	147.32 ± 42.75	119.11 ± 11.49	145.82 ± 21.75	0.2	2.51
Leucine	168.12 ± 42.43^b^	149.21 ± 17.18^b^	220.11 ± 38.67^a^	0.01	9.01
Lysine	151.64 ± 51.86^b^	145.41 ± 39.91^b^	226.75 ± 58.53^a^	0.03	6.39
Methionine	88.71 ± 17.37	86.76 ± 16.96	88.33 ± 14.36	0.98	0.03
3.Methylhistidine	15.76 ± 9.86	9.66 ± 5.84	15.48 ± 4.37	0.29	1.89
Ornithine	69.51 ± 21.47	51.64 ± 10.33	80.41 ± 20.64	0.05	5.11
Phenylalanine	62.55 ± 14.52	62.28 ± 11.03	72.33 ± 9.43	0.29	1.87
Proline	291.76 ± 77.75^b^	338.51 ± 15.55^ab^	385.78 ± 51.81^a^	0.03	5.92
Serine	164.08 ± 51.81	174.83 ± 26.26	168.26 ± 14.95	0.95	0.19
Taurine	17.53 ± 5.34^c^	56.98 ± 11.52^a^	26.06 ± 6.24^b^	0.00	51.65
Threonine	153.46 ± 31.72^b^	419.71 ± 118.92^a^	205.45 ± 71.29^b^	0.00	23.62
Tryptophan	9.79 ± 2.69	9.93 ± 2.29	10.71 ± 2.67	0.93	0.31
Tyrosine	124.43 ± 30.24^a^	92.88 ± 24.69^b^	148.20 ± 14.89^a^	0.01	10.59
Valine	288.11 ± 62.5	339.15 ± 49.32	362.81 ± 57.39	0.11	3.64
TAA	4550.46 ± 988.44	5191.16 ± 401.62	5047.21 ± 422.35	0.25	0.19
EAA	1118.41 ± 226.15	1377.53 ± 159.53	1382.49 ± 209.90	0.07	4.54
BCAA	603.49 ± 145.87	607.43 ± 74.07	728.85 ± 110.52	0.14	3.13
LNAA	1091.23 ± 203.22	1324.96 ± 152.79	1303.89 ± 165.18	0.08	4.36

*Data analysed via one-way ANOVA with *post hoc* Fisher’s least significant difference test. Values are represented as mean ± standard deviation of three batches of each milk type. ^a-c^Values without a common superscript are different (FDR < 0.05) from each other. FDR, false discovery rate, TAA, total amino acid; EAA, essential amino acid; BCAA, branched chain amino acid; LNAA, large neutral amino acid.

### 3.4. Plasma non-polar metabolites

A total of 438 features were obtained during the initial lipidomic data analysis process. After filtration and removal of background noise, 324 features were identified and used for subsequent statistical analyses.

The PCA model separated plasma lipid metabolite profiles between milk treatments, although some overlap between milk treatments was observed (Supplementary Figure S2B). A validated PLS-DA model (Q2 = 0.86 and CV-ANOVA *p*-value = <0.001) with a good fit showed clear and robust separation of plasma lipids between milk treatments (Figures 2B,D).

The relative intensity of 82 lipids was significantly different (FDR < 0.05) between milk treatments and contributed the most to the separation between milk treatments (VIP > 1) (Table 5). Therefore, these selected lipids were used further for pair-wise comparison between milk groups (Table 5).

**Table 5:** Lipids with a significant difference in relative intensities in plasma of pigs fed bovine, caprine, or ovine milk treatment ^+^.

Lipids	VIP	*F*-value	FDR	Log_2_fold change		
				Ovine vs. Bovine	Ovine vs. Caprine	Caprine vs. Bovine
DG 48:6	1.23	12.78	<0.01	−0.44	0.19	−0.62
DG 48:9	1.10	5.10	0.05	0.98	0.59	0.39
DG 49:6	1.42	11.53	<0.01	−0.68	0.27	−0.95
LPC 18:1/0:0	1.16	4.87	0.05	−0.45	0.08	−0.53
LPC 20:4/0:0	1.13	4.99	0.05	0.78	0.26	0.52
PC 30:0	1.34	11.04	<0.01	−0.50	0.34	−0.84
PC 31:0	1.67	25.81	<0.01	−0.33	0.66	−0.99
PC 31:2	1.09	5.20	0.04	−0.70	0.61	−1.31*
PC 32:0	1.15	6.67	0.02	−0.36	0.40	−0.75
PC 32:1	1.64	29.92	<0.01	−0.73	0.07	−0.79
PC 33:1	1.28	10.50	0.01	−0.47	0.43	−0.90
PC 33:3	1.27	9.29	0.01	−1.14	1.04	−2.18*
PC 34:1	1.37	8.13	0.01	−0.38	0.07	−0.45
PC 34:3	1.69	31.70	<0.01	−0.14	0.97	−1.11*
PC 35:0	1.13	6.12	0.03	−0.43	0.34	−0.77
PC 35:3	1.38	17.03	<0.01	−0.08	0.62	−0.70
PC 35:4	1.31	11.32	<0.01	−0.14	0.51	−0.65
PC 35:5	1.27	8.82	0.01	−0.77	0.74	−1.52*
PC 35:6	1.34	14.32	<0.01	−0.06	0.72	−0.78
PC 36:1	1.51	5.62	0.04	−0.84	−0.1	−0.74
PC 16:0_20:4	1.11	5.56	0.04	0.45	0.23	0.21
PC 18:1_18:3	1.51	13.85	<0.01	−0.41	−0.01	−0.40
PC 36:5	1.65	33.10	<0.01	−0.89	0.67	−1.55*
PC 37:4	1.08	6.14	0.03	0.87	0.60	0.27
PC 37:5	1.51	21.12	<0.01	−0.33	0.56	−0.90
PC 38:1	1.55	19.94	<0.01	−1.71*	0.13	−1.85*
PC 38:2	1.06	6.10	0.03	−0.27	0.33	−0.60
PC 38:4	1.28	5.51	0.04	0.97	0.28	0.70
PC 39:6	1.01	10.59	0.01	0.82	1.07*	−0.25
PC O-32:1	1.39	6.86	0.02	−0.75	0.01	−0.76
PC O-36:1	1.24	5.33	0.04	−1.05*	−0.27	−0.78
PC O-37:10	1.49	16.89	<0.01	−0.57	0.11	−0.68
PC O-39:10	1.47	16.16	<0.01	−0.22	−0.05	−0.17
PC O-39:9	1.17	6.08	0.03	−0.16	0.04	−0.20
PC O-41:10	1.45	6.08	0.03	−0.32	−0.03	−0.29
PE 36:1	1.25	5.51	0.04	−1.6	−0.03	−1.57
SM 32:1;2O	1.43	8.12	0.01	−0.84	−0.08	−0.76
SM 33:1;2O	1.20	7.08	0.02	−0.69	0.29	−0.98
SM 36:1;2O	1.46	9.94	0.01	−0.83	−0.07	−0.76
SM 38:7;3O	1.35	9.04	0.01	−0.57	0.11	−0.69
SM 39:7;2O	1.41	7.49	0.02	0.80	0.36	0.44
SM 40:2;2O	1.21	4.95	0.05	−0.48	0.18	−0.66
SM 41:2;2O	1.08	5.57	0.04	−0.62	1.02	−1.64*
TG 14:0_15:0_18:1	1.00	8.11	0.01	0.04	0.94	−0.90
TG 14:0_16:0_18:0	1.27	8.92	0.01	−0.64	0.55	−1.19*
TG 14:0_18:1_20:4	1.07	13.16	<0.01	1.08*	1.16*	−0.08
TG 15:0_15:0_21:4	1.02	12.31	<0.01	0.80	1.06*	−0.26
TG 15:0_17:0_17:0	1.14	7.87	0.01	−0.35	0.52	−0.87
TG 16:0_16:0_15:1	1.10	7.99	0.01	−0.17	0.60	−0.77
TG 16:0_16:0_16:0	1.37	12.27	<0.01	−0.52	0.37	−0.89
TG 16:0_16:0_17:0	1.04	6.19	0.03	−0.32	0.67	−0.99
TG 17:0_17:0_17:3	1.21	10.73	0.01	1.05	1.53*	−0.48
TG 18:1_18:1_16:3	1.05	12.09	<0.01	0.83	0.85	−0.02
TG 45:0	1.11	8.36	0.01	−0.26	0.93	−1.18*
TG 46:0	1.24	8.53	0.01	−0.59	0.59	−1.18*
TG 47:0	1.23	10.93	<0.01	−0.38	0.85	−1.23*
TG 48:4;1O	1.18	7.93	0.01	−0.38	0.73	−1.11*
TG 49:1	1.01	8.06	0.01	−0.02	0.80	−0.82
TG 49:3	1.02	11.07	<0.01	0.68	1.24*	−0.56
TG 49:4	1.06	11.11	<0.01	1.22*	1.86*	−0.63
TG 50:1	1.10	5.89	0.03	−0.18	0.33	−0.51
TG 51:5	1.32	14.19	<0.01	1.50*	1.81*	−0.31
TG 52:4	1.00	9.81	0.01	0.71	0.66	0.04
TG 52:6	1.06	13.75	<0.01	1.03*	1.24*	−0.21
TG 53:4	1.26	5.91	<0.01	0.84	0.83	0.02
TG 53:5	1.37	28.38	<0.01	1.05*	1.11*	−0.06
TG 53:6	1.34	17.57	<0.01	1.17*	1.83*	−0.66
TG 54:4	1.06	5.32	0.04	0.59	0.37	0.22
TG 54:5	1.13	12.03	<0.01	0.88	0.67	0.21
TG 54:6	1.13	13.72	<0.01	0.85	0.83	0.02
TG 54:7	1.05	12.84	<0.01	1.02*	1.12*	−0.10
TG 55:5	1.48	29.31	<0.01	1.07*	0.73	0.35
TG 55:6	1.14	13.37	<0.01	0.97	0.93	0.04
TG 55:7	1.47	13.84	<0.01	1.14*	1.12*	0.03
TG 56:5	1.29	9.68	0.01	0.60	0.23	0.37
TG 56:6	1.33	15.97	<0.01	0.85	0.50	0.35
TG 56:7	1.08	10.68	0.01	0.71	0.59	0.12
TG 56:8	1.13	13.65	<0.01	1.17*	1.04*	0.12
TG 58:6	1.14	8.33	0.01	0.84	0.82	0.02
TG 58:7	1.02	6.54	0.02	0.39	0.28	0.11
TG 58:8	1.23	9.99	0.01	0.85	0.43	0.42
TG 58:9	1.17	11.38	<0.01	1.29*	0.92	0.37

^+^VIP value >1 from the partial least squares discriminant analysis model was used to select the major contributing non-polar (lipids) metabolites. One-way ANOVA was used to determine the significant lipids between milk treatments (FDR < 0.05). Only lipids that satisfied multivariate and ANOVA analysis criteria are shown. *Lipids with significant fold change > ±1 (FDR < 0.05 using *t*-test) between treatments. FDR, false discovery rate; VIP, variable importance in projection; DG, diglycerides; LPC, lysophosphatidylcholine; PC, phosphatidylcholine; PC-O, ether-linked phosphatidylcholine; PE, phosphatidylethanolamine; SM, sphingomyelin; TG, triglycerides.

The relative intensity FC of 11 lipids (TG) in the ovine milk group was significantly higher than in the bovine milk group (Table 5). In contrast, the relative intensity FC of two phospholipids [phosphatidylcholine (PC) 38:1, PC O-36:1] in the ovine milk group was significantly lower than in the bovine milk group (Table 5). The relative intensity FC of 13 lipids [1 phospholipid (PC 39:6) and 12 TG] in the ovine milk group was significantly higher (log_2_FC > ±1 and FDR < 0.05) than in the caprine milk group (Table 5). Conversely, the relative intensity FC of 12 lipids [6 phospholipids, 1 sphingolipid (sphingomyelin (SM) 41:2;2O), 5 TG] in the caprine milk group was significantly lower than in the bovine milk group (Table 5).

Overall, the relative intensity pattern of plasma unsaturated TG obtained with the ovine milk treatment was distinct from caprine and bovine milk treatments, whereas the intensity pattern of PC obtained with the bovine milk treatment was distinct from those from caprine and ovine milk treatments (Figure 3B).

### 3.5. Correlation between milk nutrient intakes and plasma metabolites levels

As pigs were fed different volumes of milk in their last meal to match protein intake (2 g/kg BW; Supplementary Table S1), this adjustment resulted in different amounts of nutrients consumed by pigs between milk groups (Tables 1, 2). Hence, analysis was performed to correlate nutrient intakes with plasma metabolite concentrations, regardless of milk treatments.

**Figure 4:**
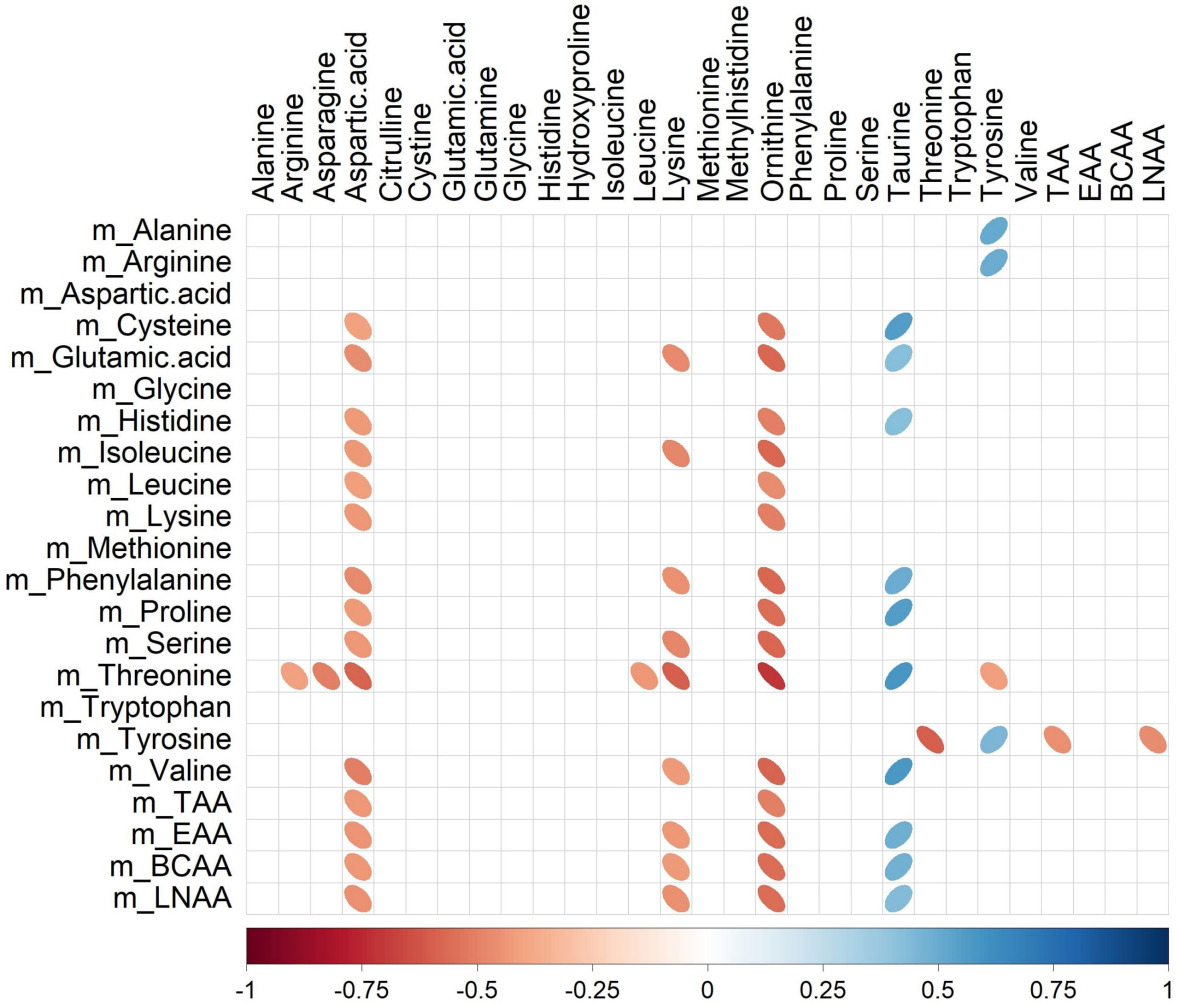
Correlation plot showing Spearman correlations between milk amino acid intake in the last meal (mg) and plasma amino acid concentrations (μmol/L) of pigs fed bovine, caprine, or ovine milk treatment. The colour of the ellipse indicates the type of correlation, i.e., blue indicates positive correlation and red indicates negative correlation. The ellipse shape indicates the magnitude of the correlation, i.e., the stronger the correlation, the flatter the ellipse. Only significant correlations (*p* < 0.05) are shown. The legend at the bottom of each correlation plot shows the correlation coefficients with their corresponding colours. The vertical axis represents amino acid intakes (preceded with the letter m), and the horizontal axis represents plasma amino acid concentrations.

Several significant correlations (*p* < 0.05) between milk amino acid intakes of the last meal (Table 1) and plasma amino acid concentrations (Table 4) were observed (Figure 4). For instance, plasma ornithine was negatively correlated with cysteine, glutamic acid, histidine, isoleucine, leucine, lysine, phenylalanine, proline, serine, threonine, and valine (rho range −0.46 to −0.71) intakes. In contrast, plasma taurine was positively correlated with cysteine, glutamic acid, histidine, phenylalanine, proline, threonine, and valine (rho range 0.42–0.58) intakes. Amongst all the correlations identified, only tyrosine intake was correlated (positively; rho = 0.45) with its peripheral blood appearance.

**Figure 5:**
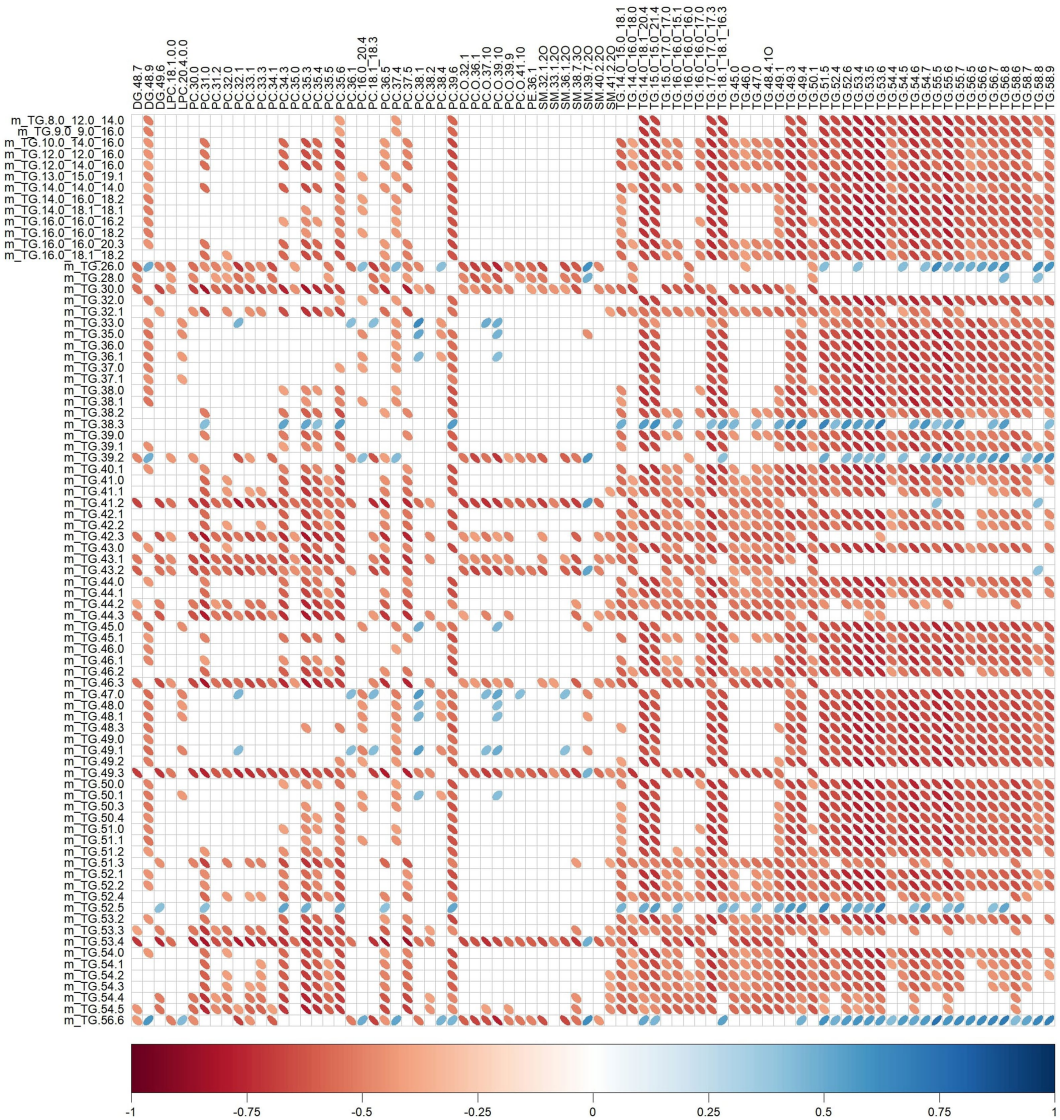
Correlation plot showing Spearman correlations between milk lipid intakes (mg) and plasma lipid relative intensities of pigs fed bovine, caprine, or ovine milk treatment. The colour of the ellipse indicates the type of correlation, i.e., blue indicates positive correlation and red indicates negative correlation. The ellipse shape indicates the magnitude of the correlation, i.e., the stronger the correlation, the flatter the ellipse. Only significant correlations (*p* < 0.05) are shown. The legend at the bottom of each correlation plot shows the correlation coefficients with their corresponding colours. The vertical axis represents milk lipid intakes (preceded with the letter m), and the horizontal axis represents plasma lipid relative intensities.

Significant relationships between milk lipid intakes (Table 2) and plasma lipid relative intensities (Table 5) were also observed (Figure 5). For instance, plasma SM 32:1,2O was negatively correlated with TG.26.0, TG.28.0, TG.30.0, TG.39.2, TG.41.2, TG.42.3, TG.43.1, TG.43.2, TG.46.3, TG.49.3, TG.53.4, TG.56.6 (rho range −0.43 to −0.69) intakes.

## 4. Discussion

This study is the first to explore the effects of feeding whole milk from bovine, caprine or ovine species on plasma polar and non-polar (lipids) metabolites using an LC-MS-based metabolomics approach in young pigs as a model of the human infant. The result showed plasma polar and lipid metabolite changes in response to ruminant milk treatments. In total, 90 polar and lipid metabolites with differential relative intensities in the plasma samples discriminated bovine, caprine, and ovine milk treatments, and some of these metabolites were correlated with milk nutrient intakes in the last meal.

### 4.1. Plasma amino acid differences between milk treatments

Differences in relative intensities of polar plasma metabolites, predominantly those involved in amino acid metabolism, were observed between milk treatments. Similar results were observed when plasma amino acid concentrations were quantified. The intake of ovine milk led to higher relative intensities of proline, tyrosine, and isoleucine and amino acid derivatives (acetylcarnitine, phenyl-pyruvic acid) and higher concentrations of asparagine, leucine, lysine, proline, and tyrosine in plasma than bovine or caprine milk treatments even though protein intakes were similar. A similar observation has been reported in a study in women aged 20–40 years, showing ovine milk consumption also resulted in higher postprandial plasma concentrations of leucine, lysine, and proline compared to bovine milk (21). However, unlike the present study, the authors did not balance the milk intake as g protein/kg BW and provided the same volume of milk to all the participants. Therefore, it could be expected that the ovine milk group would have had higher concentration amino acids in the peripheral circulation, as it has a higher protein concentration than the other milk.

In the present study, pigs received similar amounts of amino acids in their last meal due to balancing the protein intake based on body weight. Correlation analysis between amino acid intake and plasma amino acid appearance showed that only tyrosine intake was positively correlated to its plasma appearance. Hence, elevated levels of plasma amino acids in response to consuming ovine milk may be due to increased and/or more rapid amino acid absorption. This hypothesis is supported by a faster rate of gastric protein emptying in pigs consuming whole raw ovine milk than in pigs fed whole raw caprine or bovine milk (11). Additionally, a positive relationship between gastric protein emptying rate and duodenal and jejunal amino acid absorption rates has been reported in pigs (27). However, in this study, many circulatory amino acids’ appearance was not different between milk treatments. This finding may be attributed to some amino acids (e.g., glutamine, tryptophan) in the diet are taken up by first-pass metabolism by splanchnic tissues (28–30). Thus, any possible differences in these amino acids profile may only be detected in the gut or liver and may not be evident in the peripheral circulation.

The higher levels of some plasma amino acids observed in response to the ovine milk treatment could influence processes such as protein synthesis and degradation, hormone synthesis, and neurotransmission. For instance, proline is involved in glutamate neurotransmitter synthesis, whereas tyrosine is involved in dopamine and norepinephrine synthesis (31–33). Hence, it is plausible that increased concentrations of some amino acids in the peripheral circulation would increase neurotransmitter production in the gut, which in turn might influence motility and secretion (34, 35) and/or brain sensory information, learning, and memory (36, 37). For instance, we recently reported that young pigs consuming ovine milk had increased neurotransmitter glutamate and serotonin receptor gene expression in the striatum (38).

In this study, correlations were observed between plasma amino acids and amino acids intake, regardless of the milk groups. Specifically, taurine showed a positive correlation, whilst ornithine correlated negatively with amino acids intake. Although milk taurine amount was not quantified in this study, previous studies have demonstrated a positive association between dietary taurine and plasma taurine levels (39, 40), suggesting that dietary amino acid intake can influence peripheral amino acid concentrations. Furthermore, studies have indicated a positive link between sulphur amino acids, like cysteine (a taurine precursor), and plasma taurine levels (41–43), supporting the findings of this study. The reason for the negative correlation between ornithine and amino acid intake is not well understood. However, it has been observed in other study that dietary protein concentration is negatively associated with ornithine levels in the blood (44), which might be due to competition between ornithine and other dietary amino acids for absorption in the intestine, affecting the appearance of ornithine in the bloodstream. It is important to note that plasma amino acid levels can be influenced by factors such as dietary uptake, tissue metabolism, *de novo* synthesis, and the gut microbiome (45), which partially might explain the observed correlations between amino acids intake and plasma amino acids in this study.

### 4.2. Plasma lipid differences between milk treatments

Differences in plasma TG, PC, SM, LPC, and diglyceride (DG) relative intensities were observed between milk treatments. Pigs fed bovine milk had higher relative intensities of plasma lipids, except for unsaturated TG than those fed ovine or caprine milk. A study involving four-week-old pigs fed bovine or caprine milk showed that their plasma lipid profiles had predominantly TG, PC, SM, DG, and ceramides (46). However, the authors did not compare plasma lipids between ruminant milk treatments, and instead, compared the plasma lipid profile of bovine or caprine milk with human breast milk and showed differences in the lipid profiles.

In the present study, plasma saturated and unsaturated TG relative intensities differed between milk treatments. Pigs fed ovine milk showed higher relative intensities of plasma unsaturated TG than that of caprine or bovine milk, possibly due to faster gastric lipid emptying (11). In addition, studies in rats showed that unsaturated fatty acids were more efficiently absorbed than saturated fatty acids in the small intestine (47, 48), supporting the appearance of higher levels of unsaturated TG in the peripheral circulation following consumption of ovine milk. In contrast, pigs fed bovine milk showed higher relative intensities of plasma saturated TG than that of caprine or ovine milk might be due to the higher saturated TG intake of the bovine milk-fed pigs. Furthermore, studies have shown that absorption of TG is efficient when lipids intake is high (49, 50), supporting higher levels of saturated TG in the plasma of pigs fed with bovine milk.

Negative correlations were observed between specific TG intake and its appearance in circulation in the current study. The complex digestion and absorption patterns of lipids might help to explain this phenomenon. For example, TG undergo hydrolysis of fatty acids and monoacylglycerol in the small intestinal lumen. These metabolites then enter enterocytes and are incorporated into TG, which are packaged into chylomicrons that are transported via the lymph to the systemic circulation (51). Therefore, the lipid species ingested might have been broken down and assembled into different lipid species that appeared in the peripheral circulation. It is important to note that due to the nature of the milk lipid data (i.e., relative intensity data), it was challenging to determine the exact intake of lipids in the milk. Hence, the milk lipid intake data presented in the current study might not accurately represent the actual lipid intakes of the pigs.

The changes in relative intensities of lipids in the peripheral circulation could influence lipid synthesis, cell signalling, and the structural components of cell membranes. For example, phospholipids are precursors of the neurotransmitter acetylcholine (52, 53) which regulates gut motility and secretions as well as learning and memory (54–56). Peripheral circulating LPC, produced from the hydrolysis of phospholipid catalysed by acyltransferases and phospholipase, serve as a transporter for long polyunsaturated fatty acids to the brain, ultimately regulating central cell signalling and cell membrane remodelling (57, 58). Isotope labelling dietary lipid molecules could help understand their appearance in the blood, and future studies should ascertain the implications of changes in plasma lipids in early postnatal life development.

### 4.3. Strengths and limitations

The main strength of this study was the application of metabolomics for analysing plasma metabolites. This approach provided a comprehensive analysis of measurable metabolites in a biological sample, which otherwise would have been missed with the targeted approach that detects only specific metabolites. Furthermore, using the LC-MS analytical platform is another strength of this study which offered high sensitivity and detection of many metabolites compared to other analytical platforms like nuclear magnetic resonance.

Another advantage of this study is using pigs as a model for human infants to understand the plasma metabolome changes in response to different milk treatments. Furthermore, considering the higher similarity between pigs and humans in terms of gut and brain physiology, anatomy, and development than in other non-primate models like rodents (59, 60), the results can be translated for future studies with human infants, hence increasing applicability.

There are also some limitations which should be considered in interpreting findings. The use of metabolomics helped the detection of many plasma metabolites that were significant between milk treatments. However, most features remain unidentified. Additionally, LC-MS based metabolomics approach does not provide absolute quantification of metabolites, unlike nuclear magnetic resonance. No control groups of pigs fed with sow’s milk showing basal expected plasma metabolic profile were compared with the pigs fed with other species’ milk. This choice was because biological samples were collected from a study aimed to compare structural changes in ruminants’ whole milk on pigs’ digestion in early postnatal life, and using pigs fed with mother’s milk was not required for the experiment.

Another limitation of this study was the use of a single time-point for evaluating the peripheral blood plasma metabolome profile in response to milk treatments, making it difficult to ascertain whether the observed changes were an immediate diet effect or an early postnatal developmental effect. Furthermore, only male pigs were used to understand the changes in plasma metabolite relative intensities. However, a study has shown that the plasma metabolome of children with autism spectrum disorder was sex-specific (61). Hence, a comparative analysis of the plasma metabolome between male and female pigs would have been informative, but it would have required more pigs per treatment.

## 5. Conclusion

In conclusion, this study demonstrated that the consumption of whole milk from various ruminant species (bovine, caprine, and ovine) affects the metabolite profiles in the circulatory plasma of young pigs, serving as a model for human infants. The analysis of plasma metabolites using LC-MS-metabolomics revealed significant alterations, particularly in lipid profiles, between milk treatments. Specifically, the study found that the relative levels of unsaturated TG were higher in response to ovine milk consumption, whereas saturated TG levels were higher with bovine milk consumption. Furthermore, the correlation analysis highlighted a relationship between some nutrients’ intake and their appearance in the peripheral blood circulation, indicating the influence of milk nutrients on their distribution in the bloodstream. These findings contribute valuable insights to our understanding of the metabolic effects of different ruminant milk consumption and lay the groundwork for future research investigating the potential consequences of changes in circulating metabolites during early life development.

## Data availability statement

The raw data supporting the conclusions of this article will be made available by the authors, without undue reservation.

## Ethics statement

The animal study was approved by Massey University Animal Ethics Committee (MUAEC protocol 18/97). The study was conducted in accordance with the local legislation and institutional requirements.

## Author contributions

The animal experiment was codesigned by DR, CM, and WM. DR and CM conducted the pig experiment. AJ assisted with collecting biological samples, conducted the laboratory experiments and data analysis, and wrote the original draft. CM and NR helped in structuring the paper and critically reviewed the paper. KF helped with the metabolomics data analyses. CG prepared the samples for plasma amino acid analysis by HPLC. CM, NR, KF, WY, JM, WM, RD, and CG advised and critically reviewed the paper. WM and NR sourced the funding for the study. All authors contributed to the article and approved the submitted version.

## Funding

AJ was supported by a PhD Fellowship from the Riddet Institute through funding provided by the NZ Ministry of Business, Innovation and Employment (MBIE), Smarter Lives: New opportunities for dairy products across the lifespan Grant (C10X1706). Funding for this study was provided by MBIE through two programmes: the New Zealand Milk Means More (NZ3M) programme and the Smarter Lives Grant programme. CM, WY, KF, and WM are also supported by both MBIE grants. NR was supported by the Smarter Lives grant from 2018 to 2019 and the NZ3M grant.

## Conflict of interest

The authors declare that the research was conducted in the absence of any commercial or financial relationships that could be construed as a potential conflict of interest.

The reviewer CB declared a past co-authorship with the author RD to the handling editor.

## Publisher’s note

All claims expressed in this article are solely those of the authors and do not necessarily represent those of their affiliated organizations, or those of the publisher, the editors and the reviewers. Any product that may be evaluated in this article, or claim that may be made by its manufacturer, is not guaranteed or endorsed by the publisher.

## Figures and Tables

**Table:** 

LC-MS	Liquid-chromatography-mass spectrometry
RP-HPLC	Reversed-phase high-performance liquid chromatography
PND	Postnatal day
BW	Body weight
HPLC	High-performance liquid chromatography
ANOVA	Analysis of variance
CV-ANOVA	Analysis of variance of cross-validated residuals
PCA	Principal component analysis
PLS-DA	Partial least squares discriminant analysis
VIP	Variable important for projection
FC	Fold change
FDR	False discovery rate
Q^2^	Predictive ability of the model
TG	Triglycerides
DG	Diglycerides
PC	Phosphatidylcholine
SM	Sphingomyelin
LPC	Lysophosphatidylcholine
LPE	Lysophosphatidylethanolamine
TAA	Total amino acid
EAA	Essential amino acid
BCAA	Branched chain amino acid
LNAA	Large neutral amino acid
