# Supplementary material for: Metabolite profiling of peripheral blood plasma in pigs in early postnatal life fed whole bovine, caprine or ovine milk
Source: Front Nutr. 2023 Sep 26;10:1242301. doi: 10.3389/fnut.2023.1242301 (PMC10564076; doi:10.3389/fnut.2023.1242301)
Supplement: Supplementary file 1 [file Data_Sheet_1.docx]

Supplementary Material

# Supplementary Data

## Supplementary Table

**Table S1** Milk composition of bovine, caprine, and ovine raw whole milk and amounts provided in the last meal*

| **Component**** | **Raw milk** | | |  | **Intake** | | |  |  |
| --- | --- | --- | --- | --- | --- | --- | --- | --- | --- |
|  | **Bovine** | **Caprine** | **Ovine** | **F.value** | **Bovine** | **Caprine** | **Ovine** | **F.value** |  |
| Dry matter (g) | 13.10± 0.20^b^ | 11.23 ± 0.31^c^ | 17.6 0± 0.10^a^ | 672.58 | 34.38 ± 4.80^a^ | 36.15 ± 4.15^a^ | 27.82 ± 3.07^b^ | 9.31 |  |
| Protein (g) | 3.61 ± 0.07^b^ | 3.16 ± 0.11^c^ | 6.27 ± 0.08^a^ | 1153.95 | 9.50 ± 1.33 | 10.20 ± 1.17 | 9.93 ± 1.10 | 0.69 |  |
| Fat (g) | 4.06 ± 0.27^b^ | 3.22 ± 0.17^c^ | 6.31 ± 0.09^a^ | 202.08 | 10.66 ± 1.49 | 10.39 ± 1.19 | 9.97 ± 1.10 | 0.59 |  |
| Lactose (g) | 4.56 ± 0.09^a^ | 3.91 ± 0.07^c^ | 4.16 ± 0.06^b^ | 55.11 | 11.97 ± 1.67^a^ | 12.61 ± 1.45^a^ | 6.59 ± 0.73^b^ | 48.41 |  |
| Gross energy (kcal) | 75.80 ± 1.84^b^ | 61.11 ± 1.23^c^ | 107.50 ± 0.27^a^ | 1020.88 | 198.95 ± 27.78 | 196.63 ± 22.58 | 169.90 ± 18.75 | 3.83 |  |

*Data analysed via one-way ANOVA with post hoc Fisher’s least significant difference test. **Values are represented as mean ± standard deviation of three batches of each milk type and nutrient intake of pigs. a-c Values in the same row without a common superscript are different (FDR <0.05) from each other*.*

**Table S2** Lipids with a significant difference in relative intensities between the bovine (n=3), caprine (n=3), and ovine (n=3) milk samples**^+^**

| **Lipid** | **F.value** | **FDR** | **Log_2_fold change** | | |
| --- | --- | --- | --- | --- | --- |
|  |  |  | **Ovine vs Bovine** | **Ovine vs Caprine** | **Caprine vs Bovine** |
| TG 8:0_12:0_14:0 | 16.14 | <0.01 | 0.61 | 1.1* | -0.49 |
| TG 9:0_9:0_16:0 | 14.52 | <0.01 | 0.59 | 1.09* | -0.49 |
| TG 10:0_14:0_16:0 | 10.69 | 0.01 | 0.7 | 0.65 | 0.05 |
| TG 12:0_12:0_16:0 | 12.77 | 0.01 | 0.74 | 0.76 | -0.02 |
| TG 12:0_14:0_16:0 | 9.57 | 0.02 | 0.67 | 0.58 | 0.09 |
| TG 13:0_15:0_19:1 | 32.82 | <0.01 | 0.66 | 1.18* | -0.52 |
| TG 14:0_14:0_14:0 | 6.28 | 0.04 | 0.68 | 0.49 | 0.19 |
| TG 14:0_16:0_18:2 | 18.47 | <0.01 | 0.37 | 0.82 | -0.45 |
| TG 14:0_18:1_18:1 | 12.05 | 0.01 | 0.33 | 0.71 | -0.37 |
| TG 16:0_16:0_16:2 | 22.4 | <0.01 | 0.49 | 0.83 | -0.34 |
| TG 16:0_16:0_18:2 | 8.6 | 0.02 | 0.33 | 0.7 | -0.36 |
| TG 16:0_16:0_20:3 | 8.16 | 0.02 | 0.76 | 0.8 | -0.04 |
| TG 16:0_18:1_18:2 | 10.9 | 0.01 | 0.92 | 0.9 | 0.02 |
| TG 26:0 | 10.66 | 0.01 | 1.99* | 1.99* | 0.01 |
| TG 28:0 | 15.64 | <0.01 | 1.62* | 1.92* | -0.3 |
| TG 30:0 | 19.47 | <0.01 | 1.43* | 1.09* | 0.34 |
| TG 32:0 | 19.9 | <0.01 | 0.8 | 1.34* | -0.53 |
| TG 32:1 | 6.92 | 0.03 | 1.12 | 0.5 | 0.62 |
| TG 33:0 | 6.85 | 0.03 | 0.81 | 1.6* | -0.78 |
| TG 35:0 | 21.2 | <0.01 | 0.65 | 1.31* | -0.66 |
| TG 36:0 | 20.93 | <0.01 | 0.27 | 0.83 | -0.56 |
| TG 36:1 | 40.76 | <0.01 | 0.48 | 1.06* | -0.59 |
| TG 37:0 | 31.84 | <0.01 | 0.65 | 1.17* | -0.53 |
| TG 37:1 | 31.96 | <0.01 | 0.89 | 1.46* | -0.56 |
| TG 38:0 | 15.95 | <0.01 | 0.45 | 0.81 | -0.36 |
| TG 38:1 | 67.86 | <0.01 | 0.52 | 0.94 | -0.41 |
| TG 38:2 | 43.64 | <0.01 | 1.03* | 1.22* | -0.18 |
| TG 38:3 | 29.58 | <0.01 | 1.57* | 2.59* | -1.02 |
| TG 39:0 | 13.47 | 0.01 | 0.97 | 1.16* | -0.2 |
| TG 39:1 | 56.44 | <0.01 | 0.92 | 1.25* | -0.33 |
| TG 39:2 | 29.95 | <0.01 | 1.79* | 2.04* | -0.25 |
| TG 40:1 | 22.56 | <0.01 | 0.71 | 0.9 | -0.19 |
| TG 41:0 | 12.58 | 0.01 | 0.98 | 0.98 | 0 |
| TG 41:1 | 7.8 | 0.03 | 1.04* | 0.86 | 0.18 |
| TG 41:2 | 73.84 | <0.01 | 2.57* | 1.74* | 0.83 |
| TG 42:1 | 14.04 | 0.01 | 0.89 | 0.62 | 0.27 |
| TG 42:2 | 33.49 | <0.01 | 0.97 | 0.52 | 0.45 |
| TG 42:3 | 56.1 | <0.01 | 1.36* | 0.92 | 0.44 |
| TG 43:0 | 15.79 | 0.01 | 0.83 | 0.96 | -0.12 |
| TG 43:1 | 19.57 | <0.01 | 1.36* | 1.4* | -0.04 |
| TG 43:2 | 9.3 | 0.02 | 1.63* | 1.81* | -0.18 |
| TG 44:0 | 7.07 | 0.03 | 0.4 | 0.57 | -0.16 |
| TG 44:1 | 20.58 | <0.01 | 0.89 | 0.51 | 0.39 |
| TG 44:2 | 36.21 | <0.01 | 1.22* | 0.49 | 0.72 |
| TG 44:3 | 14.13 | 0.01 | 1.32* | 0.8 | 0.52 |
| TG 45:0 | 10.14 | 0.02 | 0.41 | 1.06* | -0.65 |
| TG 45:1 | 14.49 | 0.01 | 0.8 | 0.98 | -0.18 |
| TG 46:0 | 27.35 | <0.01 | 0.18 | 0.75 | -0.57 |
| TG 46:1 | 11.48 | 0.01 | 0.38 | 0.64 | -0.26 |
| TG 46:2 | 14.49 | 0.01 | 0.88 | 0.45 | 0.43 |
| TG 46:3 | 48.12 | <0.01 | 1.39* | 0.52 | 0.86 |
| TG 47:0 | 25.54 | <0.01 | 0.33 | 1.08* | -0.74 |
| TG 48:0 | 17.66 | <0.01 | 0.13 | 0.71 | -0.59 |
| TG 48:1 | 32.81 | <0.01 | 0.26 | 0.88 | -0.62 |
| TG 48:3 | 12.77 | 0.01 | 0.56 | 0.95 | -0.4 |
| TG 49:0 | 30.85 | <0.01 | 0.44 | 0.99 | -0.55 |
| TG 49:1 | 37.36 | <0.01 | 0.54 | 1.25* | -0.71 |
| TG 49:2 | 10.82 | 0.01 | 0.63 | 1.12* | -0.48 |
| TG 49:3 | 12.52 | 0.01 | 1.71* | 1.63* | 0.09 |
| TG 50:0 | 7.07 | 0.03 | 0.12 | 0.46 | -0.33 |
| TG 50:1 | 21.21 | <0.01 | 0.1 | 0.69 | -0.6 |
| TG 50:3 | 15.89 | <0.01 | 0.73 | 1.12* | -0.39 |
| TG 50:4 | 9.02 | 0.02 | 0.91 | 1.28* | -0.37 |
| TG 51:0 | 11.14 | 0.01 | 0.72 | 1.06* | -0.35 |
| TG 51:1 | 37.65 | <0.01 | 0.62 | 1.02* | -0.41 |
| TG 51:2 | 39.24 | <0.01 | 0.94 | 1.2* | -0.26 |
| TG 51:3 | 14.03 | 0.01 | 1.21* | 1.29* | -0.09 |
| TG 52:1 | 23.25 | <0.01 | 0.44 | 0.65 | -0.21 |
| TG 52:2 | 12.17 | 0.01 | 0.58 | 0.78 | -0.2 |
| TG 52:4 | 11.77 | 0.01 | 1.15 | 1.37* | -0.21 |
| TG 52:5 | 13.43 | 0.01 | 1.49 | 2.39* | -0.9 |
| TG 53:2 | 24.82 | <0.01 | 0.81 | 0.86 | -0.04 |
| TG 53:3 | 10.2 | 0.02 | 1.24 | 1.27* | -0.03 |
| TG 53:4 | 16.83 | 0.01 | 1.83* | 1.53* | 0.3 |
| TG 54:0 | 6.42 | 0.04 | 0.82 | 0.85 | -0.03 |
| TG 54:1 | 32.68 | <0.01 | 0.93 | 0.85 | 0.08 |
| TG 54:2 | 27.01 | <0.01 | 1.03* | 0.81 | 0.22 |
| TG 54:3 | 15.14 | 0.01 | 1.04* | 0.7 | 0.33 |
| TG 54:4 | 9.49 | 0.02 | 1.23* | 0.85 | 0.38 |
| TG 54:5 | 17.2 | <0.01 | 1.26* | 0.99 | 0.27 |
| TG 56:6 | 68 | <0.01 | 2.66* | 2.62* | 0.04 |

+VIP value >1 from the PLS-DA model was used to select the major contributing lipids. One-way ANOVA was used to determine the significant lipid relative intensities between milk types (FDR < 0.05). Only the polar metabolites that satisfied the multivariate and ANOVA analysis criteria are shown. *Lipids with significant log2 fold change > ± 1 (FDR < 0.05 using t-test) between milk. VIP, variable importance in projection; FDR, false discovery rate; TG, triglyceride

## Supplementary Figure


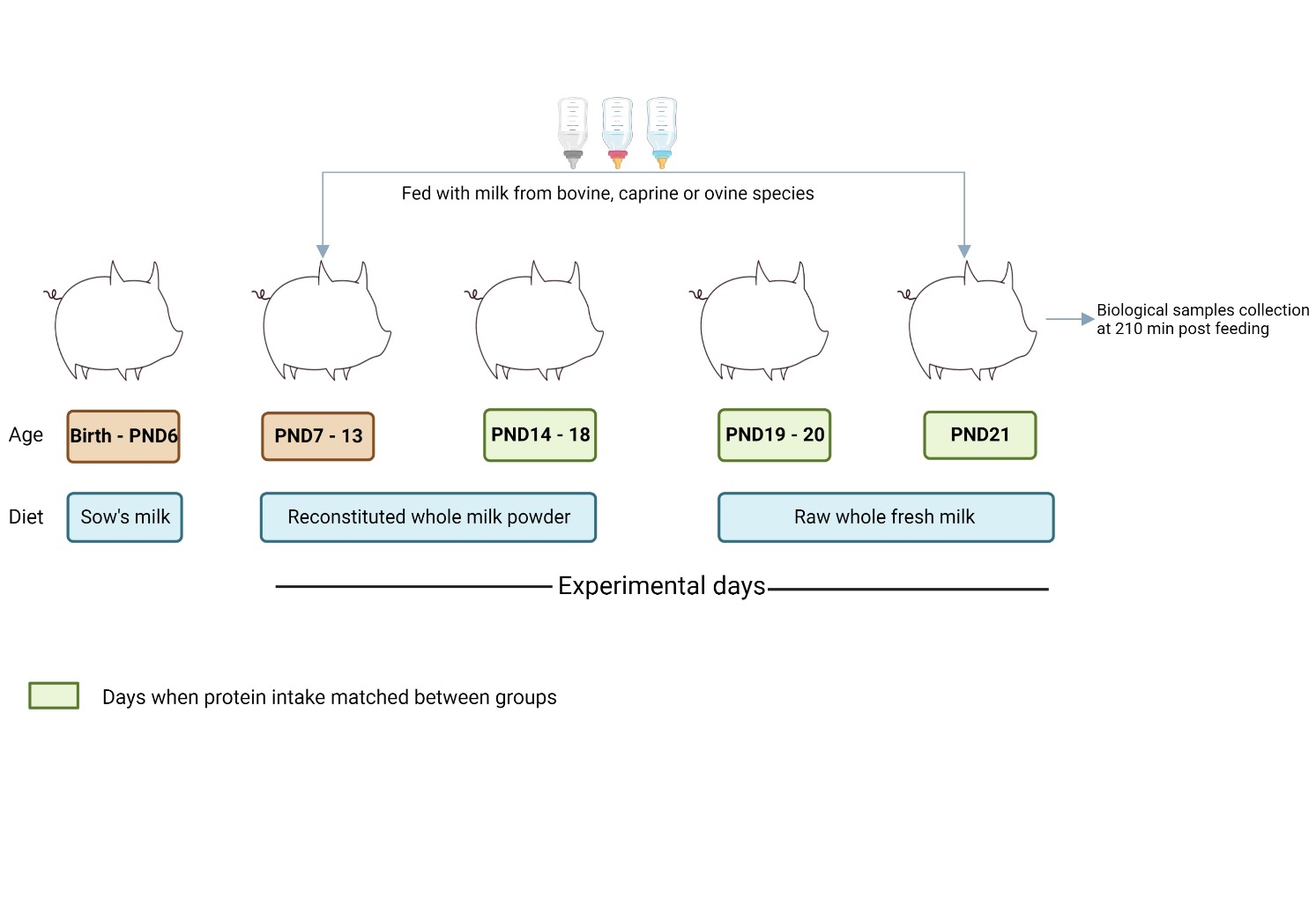


**Figure S1** Overview of the *in vivo* pig study. PND, postnatal day. Figure created with biorender.com


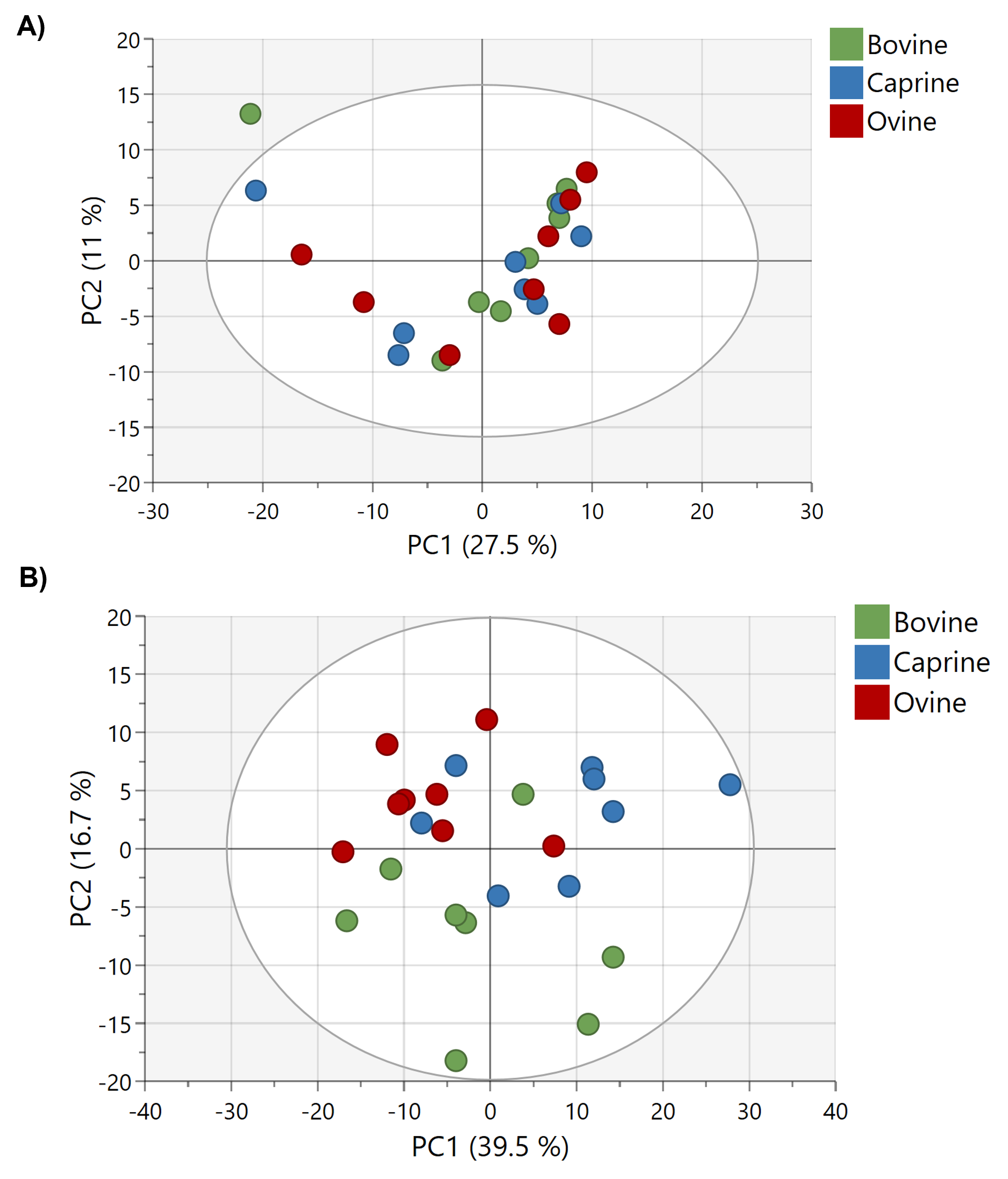


Figure S2 Principal component analysis (PCA) score plot showing A) polar B) non-polar (lipids) metabolites differences in plasma of pigs following bovine, caprine, or ovine milk treatments. The first two principal components are plotted. Percentages of variation explained by each principal component are indicated along the axes. PC, principal component
